# Reconceptualizing learning engagement: evidence for a context-sensitive structure in STEM education

**DOI:** 10.3389/fpsyg.2025.1649744

**Published:** 2025-11-11

**Authors:** Eric Trevor McChesney, Christian D. Schunn, Gerard Dorvè-Lewis, Allison Godwin, Linda DeAngelo

**Affiliations:** 1 Learning Research and Development Center, University of Pittsburgh, Pittsburgh, PA, United States; 2 Department of Psychology, University of Pittsburgh, Pittsburgh, PA, United States; 3 Department of Educational Foundations, Organizations, and Policy, University of Pittsburgh, Pittsburgh, PA, United States; 4 R.F. Smith School of Chemical and Biomolecular Engineering, Cornell University, Ithaca, NY, United States

**Keywords:** engagement, learning, STEM – science technology engineering mathematics, ABC model, higher education, factor structure, measurement, disengagement

## Abstract

Understanding student engagement as a psychological construct remains a persistent challenge in educational psychology, particularly in higher education STEM contexts. Traditional models distinguish engagement into behavioral, cognitive, and affective dimensions, yet often overlook how the structure of engagement may be shaped by contextualized learning activities. This study introduces and tests a novel, activity space-based model of engagement, hypothesizing that behavioral and cognitive engagement are organized by the specific academic environments in which they occur (e.g., lectures, exams, projects, recitations). Applying new engagement survey instruments that were iteratively developed to be contextually meaningful, we first present an exploratory factor analysis applied to 1,176 students from two different courses and institutions. Then we present a confirmatory factor analysis applied to 772 students in a third course. We find that a model organized by activity contexts—rather than by behavioral and cognitive distinctions—better fits the data and generalizes across STEM disciplines. The findings challenge conventional engagement theory and support a reconceptualization of engagement as a partially context-sensitive construct. This theoretical shift has implications for psychological models of learning and for the design of more precise, equitable interventions that address varied patterns of engagement within and across STEM domains.

## Introduction

Student engagement is crucial for academic success (Fitzgerald et al., 2012; Laranjeira and Teixeira, 2024; Mayhew et al., 2016), yet its underlying psychological structure remains contested (Azevedo, 2015). This issue is particularly critical in science, technology, engineering, and mathematics (STEM), where engagement strongly influences outcomes (Grabau and Ma, 2017; Schmidt et al., 2018; Sinatra et al., 2015; Wang et al., 2016). In higher education, foundational STEM courses have high attrition rates, and evince persistent equity gaps (Beasley and Fischer, 2012; Fouad et al., 2017; Riegle-Crumb et al., 2019; Seymour and Hunter, 2019). However, efforts to define and measure learning engagement in a precise, context-sensitive manner have lagged behind its theorized importance.

Psychological models typically define engagement as a multidimensional construct comprising behavioral, cognitive, and affective components (Fredricks et al., 2004). Behavioral engagement captures goal-directed actions such as participation and effort; cognitive engagement includes metacognitive strategies and deep processing; and affective engagement reflects emotional responses such as interest or anxiety. These factors are often treated as general, trait-like constructs. However, growing evidence suggests engagement is not only multidimensional but also dynamically shaped by context (Ben-Eliyahu et al., 2018; Kahu, 2013).

In this study, we propose and evaluate a novel perspective: that behavioral and cognitive engagement are organized not solely by psychological domains, but also by activity spaces—distinct learning environments that structure goals, tools, social roles, and norms (Bakhurst, 2009; Engeström, 2000). Activity spaces such as lectures, exams, group work, and study sessions afford different forms of engagement, which may produce meaningful psychological distinctions within and across learners. If true, this would suggest that the behavioral-cognitive distinction is insufficient on its own to explain how engagement manifests in real academic contexts.

To test this hypothesis, we developed and validated an engagement scale sensitive to both psychological dimensions and activity contexts. We then examined whether student responses reflected the expected behavioral-cognitive distinction—or whether engagement patterns clustered by activity space. To do so, and to create a stronger generalizability argument for our findings across different STEM disciplines, we conducted two studies with samples drawn from different STEM courses: engineering and biology. These courses were selected due to their distinctly different structures, learning activities, and student demographic characteristics. Through exploratory and confirmatory factor analyses, we find consistent support for the activity space hypothesis.

Our research question is: What is the precise psychological structure of behavioral and cognitive engagement in undergraduate STEM learning environments? First, using exploratory factor analysis (EFA) we analyze data from an active-learning engineering course and a large-lecture genetics course. We also report pilot and replication studies (Appendix B) that support the generalizability of the findings, cognitive interviews (Appendix C), and factor invariance tests (Appendix D) that provide evidence for their validity across disciplines. Finally, confirmatory factor analysis (CFA) on a novel sample compares the model fit of a traditional behavioral-cognitive model to the fit of our new space-based model. By situating engagement within activity spaces, we aim to expand psychological models of engagement and offer more contextually grounded tools for research and intervention.

## Review of the literature

### What is engagement?

We define engagement as “the intensity of productive involvement with an activity” (Ben-Eliyahu et al., 2018, p. 87), which includes facets such as involvement, focus, participation, and persistence (Fredricks et al., 2004). We consider motivation as the semi-stable student characteristics that interact with learning contexts to produce engagement (Ben-Eliyahu et al., 2018). By contrast, engagement varies by context and is shaped by pedagogy, demographics, course structure, and task attributes (Kahu, 2013; Sinatra et al., 2015; Wang et al., 2016).

### Engagement in higher education

Due to the frequent employment of large “grain sizes” of measurement (coarse, high-level assessments of general engagement as opposed to precise, contextually appropriate assessments, see Sinatra et al., 2015), research on learning engagement in higher education often lacks the precision evident in the primary and secondary learning engagement literature. Research on engagement in K-12 contexts tends to emphasize the multidimensional nature of engagement within a psychological framework (Kahu, 2013; Zhoc et al., 2019) and focuses on teaching practices associated with positive learning outcomes (Kahu, 2013; Krause and Coates, 2008), providing pedagogically useful frameworks and tools to educators and policymakers. By contrast, much research on engagement in higher education emphasizes student behaviors at a more general level (i.e., not within a specific learning context) and highlights the student-institution relationship, where students bear primary responsibility for learning and institutions provide support and resources (Coates, 2005; Krause and Coates, 2008; Zhoc et al., 2019). Furthermore, greater tertiary student autonomy permits more varied engagement strategies than in pre-college contexts, especially in STEM (Kahn, 2014). For example, an undergraduate could learn and develop quite well via independent study and taking exams while eschewing lectures. Resultantly, professors and teaching centers desiring to increase undergraduate engagement are often left with inadequate guidance. This challenge has become especially acute in the context of growing undergraduate disengagement from learning (Al-Furaih and Al-Awidi, 2021; Allison et al., 2024; Brint and Cantwell, 2012; Cech, 2014; Chipchase et al., 2017; Greener, 2018; Hockings et al., 2008; Jang et al., 2016; McCoy, 2016; Saito and Smith, 2017) and the acceleration of this disengagement by the COVID-19 pandemic (Branchu and Flaureau, 2022; Newton and Essex, 2024; Walker and Koralesky, 2021). To provide educators with more actionable tools for assessing and bolstering learning engagement, more research is needed on its specific nature within university courses.

## Theoretical background

### B-C or B/C engagement: Trait, or context?

Within-course engagement is typically conceptualized as a multidimensional construct (Appleton et al., 2006; Ben-Eliyahu et al., 2018; Kahu, 2013; Wang et al., 2016; Zhoc et al., 2019). The affective-behavioral-cognitive (A-B-C) model is the most empirically supported engagement structure (Fredricks et al., 2004). It comprises an affective/emotional factor, a behavioral factor composed of goal-directed actions, and a cognitive factor responsible for processing, thoughts, beliefs, and interpretations of the current task. While these factors may interact, their structure remains distinct.

Affective engagement reflects students’ emotional responses to peers, instructors, the course, the discipline, and the institution. Manifestations include interest versus boredom, happiness versus sadness, anxiety versus confidence, and pleasure versus discomfort. While affective engagement plays a critical role in student well-being (Bowden et al., 2021) and the development of attitudes toward learning (Bathgate and Schunn, 2017), it is conceptually and empirically distinct from behavioral and cognitive engagement (Flowerday and Schraw, 2003). Emerging evidence (McChesney et al., 2025) demonstrates affective engagement consistently separates from behavioral and cognitive dimensions and follows a different structural logic—organized around emotional valence (positive or negative) rather than the activity space-based patterns examined in this study. In line with prior work that focuses specifically on behavioral and cognitive dimensions to enable more targeted modeling (Poellhuber et al., 2016), this study deliberately limits its scope to those two forms. This narrowed focus allows for a deeper investigation into how behavioral and cognitive engagement are structured by educational activity spaces.

The behavioral domain denotes goal-directed actions that support learning and includes on-task behavior, effort, persistence, and concentration (Fredricks et al., 2004). Manifestations include studying, class attendance, completing activities, and writing papers. These behaviors, which vary by course deliverables and structure, correlate with academic performance (Wang et al., 2016) and changes in self-efficacy and self-esteem (Bathgate and Schunn, 2017; Bowden et al., 2021).

The cognitive domain denotes mental effort in pursuit of learning outcomes, and includes thoughtfulness, depth of processing, development of goals, self-regulation, and metacognitive strategies (Fredricks et al., 2004). Manifestations include active reflection upon one’s thinking processes and learning strategies, synthesizing information from multiple sources, concept mapping, and applying conceptual structures to complex information. Cognitive engagement has been consistently observed to support academic performance (Greene, 2015; Rotgans et al., 2018).

Conceptually, there should be a strong separation between cognitive and behavioral engagement, but empirical evidence on their separation has been mixed. Generally, these factors correlate more strongly with each other than with affective engagement (Ben-Eliyahu et al., 2018; Wang et al., 2016), particularly in secondary education. This trend may reflect reciprocal causation (thinking influences action and vice-versa). But it may also reflect measurement limitations. For example, younger students may struggle to self-assess their cognition and, therefore, base responses on behavioral elements (Fredricks et al., 2004). In addition, in efforts to create instruments with validity evidence that have universal educational applicability, survey instruments are often focused on instantiations of behavioral and cognitive engagement that might poorly match the varied ways students can engage in particular contexts. In other words, better separation of cognitive and behavioral engagement might occur with instruments better tuned to each context. Such limitations highlight the need to reassess engagement not as static traits, but as dynamic, contextually structured phenomena.

### Activity spaces: Structuring learning engagement

Learning does not occur in a psychological vacuum. Rather, contemporary research acknowledges the importance of task characteristics, activity contexts, and environmental affordances in shaping learning engagement (Bedenlier et al., 2020; Branchu and Flaureau, 2022; Choi et al., 2025; Li and Xue, 2023; Limniou et al., 2022; Schmidt et al., 2018; Walker and Koralesky, 2021). We synthesize these findings through the concept of activity spaces. An activity space is a physical, social, cultural, and historical environment that forms the domain of specific behaviors (for example, studying for a quiz, listening to a lecture, or completing a team project; Bakhurst, 2009). The concept is derived from activity theory—a sociological perspective developed by Engeström and others into cultural-historical activity theory (see Engeström, 2000). Often used in psychological and educational research, activity spaces are composed of subjects (a student), their objective (studying communally for an exam), available tools (textbooks, whiteboards, other mediating artifacts), the community (several classmates and their social characteristics), norms (active information sharing), and division of labor (one student searches the textbook, another reviews notes) (Gyasi et al., 2021). Systematic reviews report STEM learning encompasses diverse activity spaces—labs, study sessions, coding tasks—each fostering distinct engagement behaviors and cognitive strategies (Gyasi et al., 2021). Research suggests engagement is shaped by activity processes (Ben-Eliyahu et al., 2018). Therefore, we argue that activity spaces do not merely modulate behavioral and cognitive engagement levels -they may shape how engagement is structured psychologically.

### Limitations of current engagement research

This study addresses key limitations in prior research on behavioral-cognitive learning engagement in higher education STEM contexts. First, there is a lack of studies that use engagement measures capable of directly studying the robustness of engagement’s structure across contexts. Some studies use a small pool of items assessing generic, decontextualized forms of engagement that could exist in any course context and thus, they struggle to capture the ways in which engagement might vary across activity spaces within a course. Other studies examine engagement within only one specific context rather than across contexts. Resultantly, different contexts are assessed with distinct item pools, which makes it difficult to determine whether differences in engagement are due to genuine contextual variation or simply differences in instrumentation. This heterogeneity prevents cross-context comparisons, limiting the generalizability of findings (Buntins et al., 2021). Large-grained instruments also frequently obscure engagement’s within-course variation, ignoring how students may engage differently across activity settings even within a single class. Addressing these shortcomings, this study employs a coherent item pool tested across multiple STEM contexts selected for their disciplinary variability (Granovskiy, 2018; see also Appendices B, D).

Second, many studies use a “large grain size” approach, measuring engagement broadly (e.g., institutional-level engagement) rather than at a fine-grained level (e.g., a student’s engagement in specific learning tasks) (Schmidt et al., 2018; Sinatra et al., 2015). However, fine-grained measures are crucial for assessing the psychological underpinnings of the learning process—a matter of particular significance for researchers, practitioners, and theorists (Azevedo, 2015).

Third, while extensive research acknowledges and examines the impact of contextual factors on engagement (Astin, 1993; Lewin, 1936; Li and Xue, 2023; Mayhew et al., 2016; Schmidt et al., 2018) this study moves beyond the common assumption that the dimensionalityof learning engagement is fixed across contexts. Previous work has productively explored context-engagement interactions along axes such as in-person versus electronic modalities (Altomonte et al., 2016), and how instructional choices shape overall engagement intensity (Zhao et al., 2021). However, few studies have tested how contextual effects differentially shape specific domains of learning engagement. Those that have (Lam et al., 2012; Şeker, 2023) typically treat contextual factors as antecedents that moderate engagement in particular domains (e.g., behavioral) without considering the internal structure within domains or the heterogeneity of context-engagement interactions that may occur there. While such approaches explain why learning engagement varies by context, this investigation goes further by examining how engagement itself is constructed -treating context as inseparable from engagement activities.

Finally, much research confounds engagement with its antecedents (e.g., motivation; see Ben-Eliyahu et al., 2018) or its consequents (e.g., academic achievement; see Kahu, 2013). This conceptual blurring obscures engagement’s structure and hinders tests of engagement’s internal structure and assessments of its predictive validity (DeVellis, 2016). This study disentangles these phenomena, clarifying engagement’s structure to help researchers generate specific insights and enable practitioners to develop more effective engagement strategies.

## Exploratory study

### Overview

A novel learning engagement instrument was developed to apply the BC engagement model in university STEM course contexts (see Appendix B for full instrument development details). It was piloted in Spring 2022 on 149 engineering students. Confirmatory factor analysis revealed the anticipated behavioral-cognitive factor structure did not fit the data, while an alternative model including in-exam cognitive engagement alongside the two expected factors did. Pursuing this unexpected finding, the scale was then iteratively revised and tested to better capture variation by activity spaces across economics (*n* = 324), organic chemistry (*n* = 198), general chemistry 2 (*n* = 346), and engineering (*n* = 810) contexts (Appendix B). To improve instrument quality, identify response errors, and enhance clarity, cognitive interviews were conducted with participants from diverse STEM fields and sociocultural backgrounds and the findings used to strengthen the scale further and contribute to its validity argument (Appendix C). We here test the finalized scale in new populations and analyze its structure via exploratory factor analyses.

### Materials and methods

#### Participants

The study took place in the Fall of 2023 at two research-intensive U. S. institutions: a rural Midwestern university in a first-year engineering programming course, and an urban Mid-Atlantic university in a second-year genetics course. The engineering course emphasized introductory data analysis and programming concepts in MATLAB and data-driven decision-making for engineering problem-solving. Instruction included a flipped learning classroom environment with team-based and paired programming problems solved in class, weekly homework assignments, quizzes, and a final team coding project. The genetics course covered gene function, mutation, evolution, and population genetics, using large lectures, smaller recitation sections, and high-stakes exams.

Survey response rates were 80% in engineering (*n* = 847) and 97% in genetics (*n* = 445). Inattentive responses were identified using an attention check item (Barge and Gehlbach, 2012) and removed, reducing the analytical sample to *n* = 774 for engineering and *n* = 402 for genetics. The demographic instrument collected detailed race/ethnicity and gender data. While acknowledging the social construction and heterogeneity of racial identities, this study aggregates some categories (e.g., Southeast Asian and South Asian as “Asian”) to highlight shared STEM marginalization experiences and to address power issues in modeling (Ladson-Billings, 2020; Nuñez et al., 2023; Wells and Stage, 2015).

In the engineering sample, 51% identified as men, 27% as women, and 22% as non-binary or preferring not to respond. Racially, the sample was 55% White, 22% Asian, and 9% Latinx. In contrast, the genetics sample was 56% women, with 56% White and 33% Asian students. These demographics align with U.S. enrollment patterns in engineering and genetics (National Center for Education Statistics, 2023). Full demographics are in Appendix Table A1.

#### Measures

##### Engagement

STEM learning engagement was assessed using sub-scales measuring cognitive/behavioral dimensions of engagement across activity spaces. The scales underwent extensive testing, refinement, and validation, including a pilot test, cognitive interviews, and two replication studies across multiple disciplines and institutions (see Appendices B, C). In total the validation effort included 1,827 students from across five courses (including organic and general chemistry, macro- and micro-economics, and engineering coding) embedded in three institutions that differed in geographic region, research intensity, selectivity, and size. Faculty instructors in these diverse contexts described their course structures (e.g., lecture, recitation, lab) and assessed item suitability for their context.

The scale included two items to assess cognitive focus during exams, three for behavioral pre-exam studying, five for cognitive engagement in lectures/classes, two for cognitive engagement in group projects, two for behavioral engagement in the same, and two for behavioral engagement in recitations. Items used a 4-point Likert-type response scale except for two behavioral items requiring numerical input. Due to the lack of meaningful differences between 4-to-11 point Likert scales (Leung, 2011), and to reduce survey fatigue, we chose a 4-point scale for most items. An example behavioral item is “I spent ___ hours with others on my team to complete the group project,” while an example cognitive item is “During the latest group assignment, I made sure to understand the plan for the project and my role in that plan.” Response options varied (e.g., agreement and frequency scales) to improve attentiveness. The full instrument and response metrics are in Appendix Tables A2, A3.

Finally, the scale has demonstrated strong measurement invariance across contexts (detailed in Appendix D). In brief, the scale was administered to a sample of 2,637 students from different STEM majors and courses (engineering coding, engineering design, general chemistry, genetics), different years in college (first year and post-first year) and from two different research-intensive institutions. Multigroup structural equation modeling provided measurement coefficients and standard errors for each item on its parent factor in each context. We used Wald’s test compare item functioning across groups and found that most items in the engagement scale were invariant in these diverse contexts and populations. This suggests considerable generalizability of both the measurement approach and the underlying phenomena in undergraduate STEM contexts.

##### Demographics

A single “select all that apply” item with 17 response options assessed racial/ethnic identities. One item measured gender identities via four response options: man, woman, non-binary/gender-queer, not listed above (please specify), and prefer not to respond.

#### Procedure

Data were collected online via Qualtrics, with demographics surveyed at the start of the Fall 2023 term and engagement at term end prior to final exams. Students received 2 participation points for starting the survey. To encourage honest responding, they were assured that instructors could not access their data, responses would be deidentified, and only aggregate findings would be reported.

#### Analysis

The data met EFA assumptions, with minimal missing data (engineering = 0.1%, genetics = 0.01%). Write-in responses were tetrachotimized based on equal observation counts. Distributional assumptions were assessed via scatter plot matrices and descriptive statistics. While some items showed non-normal skewness/kurtosis, assumptions of linearity and unimodality held. Maximum likelihood with missing values estimation was used alongside oblique promax factor rotation in Stata v.17.

### Results

The Kaiser-Meyer-Olkin (K-M-O) test indicated adequate sampling for factor analysis (0.73 engineering, 0.82 genetics) (Flora and Flake, 2017). Eigenvalue screeplots are in Appendix Figures A1, A2. The optimal factor solution was determined using (1) eigenvalues larger than 1, (2) scree plot interpretation, (3) rotated factor coherence (4) sufficient items per factor, (5) minimal cross-loadings (6) minimizing weakly loading (<0.4) items, and (7) theoretical interpretability (Flora and Flake, 2017).

In contrast to the expectations of prevailing theory, the data did not break down into a two-factor solution composed of (1) behavioral and (2) cognitive engagement. Instead, a five-factor solution emerged for engineering, consisting of (1) in-exam cognitive focus, (2) in-class cognitive strategies, (3) cognitive engagement with group assignments, (4) pre-exam studying behaviors, and (5) time spent on group assignments. An identical four-factor solution emerged for genetics except for the absence of the two group assignment factors (group assignments were not part of the course) and the addition of a behavioral factor for attending and completing activities in recitations (the engineering course did not have recitations). The maximum inter-factor correlation was 0.44 for engineering and 0.30 for genetics, indicating acceptable factor separation. Rotated factor loadings are in Table 1 with replication studies confirming the factor structure (Appendix B).

**Table 1:** Rotated factor loadings, organized by location and behavioral (black) vs. cognitive (blue) focus.

Item	Engineering design (*n* = 774)					Genetics (*n* = 402)			
	Factor 1 In-Exam Focus	Factor 2 Pre-Exam Studying	Factor 3 In-Class	Factor 4 Cog. Group Assign.	Factor 5 Group Assign. Time	Factor 1 In-Exam Focus	Factor 2 Pre-Exam Studying	Factor 3 In-Class Cognition	Factor 4 Recitation Behaviors
C14: For the most recent test, it was easy to pay attention	**0.88**					**0.89**			
C15: For the most recent test, it was easy to think clearly	**0.90**					**0.76**			
B2: While studying for the most recent exam or midterm I spent __ reorganizing my notes so the big ideas were clear		**0.72**					0.42		
B9: I started studying for the most recent exam [time categories]		**0.72**					**0.90**		
B10: I spent __ hours studying alone for the most recent exam.		**0.84**					0.40		
C5: During class, I combined different pieces of information from the course in new ways (topics from different weeks, etc.)			0.58						
C6: During class, I made pictures, diagrams, charts, or other figures to help understand the course content			0.59					0.54	
C8: I always summarized new [class/lecture] material in my own words when taking notes			**0.60**					**0.68**	
C9: When I had difficulty understanding [class/lecture] material, I marked it to come back to later			0.50					**0.70**	
C10: I focused on understanding the diagrams, charts, and figures presented in the [class/lecture]			0.48					0.59	
C11: During the latest group assignment, I made sure to understand the plan for the project and my role in that plan				**0.73**					
C12: I was able to stay mentally focused while completing my part of the project				**0.92**					
B12: I spent ___ hours with others on my team to complete the group project					**0.67**				
B13: I spent ___ hours on my own working on the team project					0.44				
B3: I have attended ___ of the recitations so far									0.48
B5: I completed ___ of the activities we were given in recitation									**0.96**
Average variance extracted	0.79	0.58	0.30	0.69	0.32	0.68	0.38	0.40	0.58

Item loadings <0.40 are not shown. Gray cells indicated expected loadings based upon factor and item content. The strongest loadings (>0.6) are bold.

Internal reliability (Cronbach’s *α*) ranged from 0.69–0.86 (engineering) and 0.56–0.79 (genetics) (Appendix Table A4). While α > 0.60 is generally acceptable for new scales (DeVellis, 2016), Cronbach’s α is downwardly biased by brief scale lengths, ordinal items, and the assumption of essential tau-equivalence (Raykov, 1997; Zumbo et al., 2007). Thus, scholars have identified lower thresholds (α > 0.50) as indicating adequate reliability for shorter scales (2–3 items) (Briggs and Cheek, 1986). Dropped versus loaded items are in Appendix Tables A5, A6.

### Discussion

Contradicting prevalent theory (Fredricks et al., 2004), the exploratory study provided evidence that behavioral-cognitive learning engagement is shaped not only by behavioral-cognitive distinctions but also by activity spaces. While findings were consistent across two STEM disciplines, small but meaningful differences emerged that were aligned with engagement affordances (e.g., group assignments) and learning contexts. Replication studies (Appendix B) and factorial invariance analysis (Appendix D) further support the potential generalizability of the hypothesized structure across STEM disciplines and courses. Replications consistently showed a clear separation of activity spaces dominated by either cognitive or behavioral engagement, with replication studies yielding the same factor structures as the main study. These results underscore the robustness of the engagement structure across STEM contexts.

## Confirmatory study

### Overview

While EFA revealed a consistent factor structure, such analysis is interpretative, and only confirmatory techniques can formally test hypothesized models. We administered the scale to a new sample and compared the fit of two alternative models: (1) the traditional cognitive-behavioral structure and (2) the activity space-based structure based on the exploratory study.

### Materials and methods

#### Participants

At a large, public, Mid-Atlantic, research university, all instructors and students in an introductory biology course were invited to participate, with students earning two extra credit points for opening each of the two surveys. The initial response rate was 69% (*n* = 883) with a final analytical sample of 772 after removing inattentive or incomplete responses. Participant demographics matched national trends (National Center for Education Statistics, 2023) (Appendix Table A1).

#### Measures

Measures were identical to those of the exploratory study with one modification. The recitation attendance and activity completion items showed limited variance and high skewness (most students attended all sessions and completed all activities). These items were dichotomized.

wherein complete attendance or participation (4) was recoded to 1, and all other non-missing responses (1–3) recoded to 0. Full items and descriptive statistics are in Appendix Table A7.

#### Procedures

Demographic and engagement data were gathered via Qualtrics at the beginning and end of the Spring 2023 term. Survey distribution, communications, and incentives were identical to those in the exploratory study.

#### Analysis

##### Data screening and estimation

Data were assessed for CFA suitability. While no variable exceeded |2| skewness or 7 kurtosis, formal skewness and kurtosis tests, Shapiro–Wilk tests (Appendix Tables A8, A9), and the Doornik-Hansen test of multivariate normality ( $\chi_{\left(22\right)}^{2}$ = 3058.2, *p* < 0.001) indicated several non-normal distributions necessitating nonparametric estimation (e.g., bootstrapping) (Brown, 2015; Flora and Flake, 2017). CFA was conducted using maximum likelihood estimation with 5,000 bootstrapped standard error iterations with resampling at the course section level to control for data clustering. Analysis took place in Stata v.18 using the *sem* package.

##### Model fit evaluation

Degree of misfit was determined by global and absolute fit indices, specifically the Comparative Fit Index (CFI), Tucker-Lewis Index (TLI), root mean squared error of approximation (RMSEA), and standardized root mean squared residual (SRMR). Following West et al. (2012), for this sample size and application, an acceptable fit was defined as CFI and TLI above 0.90, RMSEA below 0.08 and SRMR below 0.10; and good fit was defined as CFI and TLI above 0.95, RMSEA below 0.05, and SRMR below 0.08. The coefficient of determination (CD) ranges from 0 to 1 and measures variance explained with higher values indicating better explanatory models.

##### Tested models

Two hypothesized structures were tested. The first was the traditional behavioral-cognitive structure with all five behavioral items loading on the single latent behavioral factor and all six cognitive items loading on the cognitive factor. The second model tested the EFA-derived emergent structure based on spaces of engagement. Four spaces were hypothesized: pre-exam studying behavior (three items), recitation behavior (two items), in-exam focus cognition (two items), and lecture cognition (four items).

##### Model refinement

Initial models constrained latent factors orthogonally with unique item error terms and uncorrelated residuals. We evaluated model fit, then examined standardized residual matrices to detect unmodeled item-factor or factor-factor relationships and modification indices to evaluate fit improvements from added correlations. Based on these considerations, prior research, and theoretical guidance, single inter-factor correlations were added until each model was optimized.

### Results

The traditional behavioral-cognitive model failed to provide an acceptable fit to the data (CFI = 0.68; TLI = 0.60; RMSEA = 0.118, [90% CI = 0.109, 0.127, *p*-RMSEA < 0.05 = 0.000]; SRMR = 0.105; CD = 0.94). Adding covariance paths did not improve model fit, and factor loadings were weak, with one behavioral item not significantly loading onto its latent factor (Appendix Figure A3; Appendix Table A10; Brown, 2015; Flora and Flake, 2017; Kline and Little, 2023). This notable lack of fit to the data implies the traditional behavioral-cognitive model does not accurately describe the fine-grained structure of engagement.

**Figure 1:**
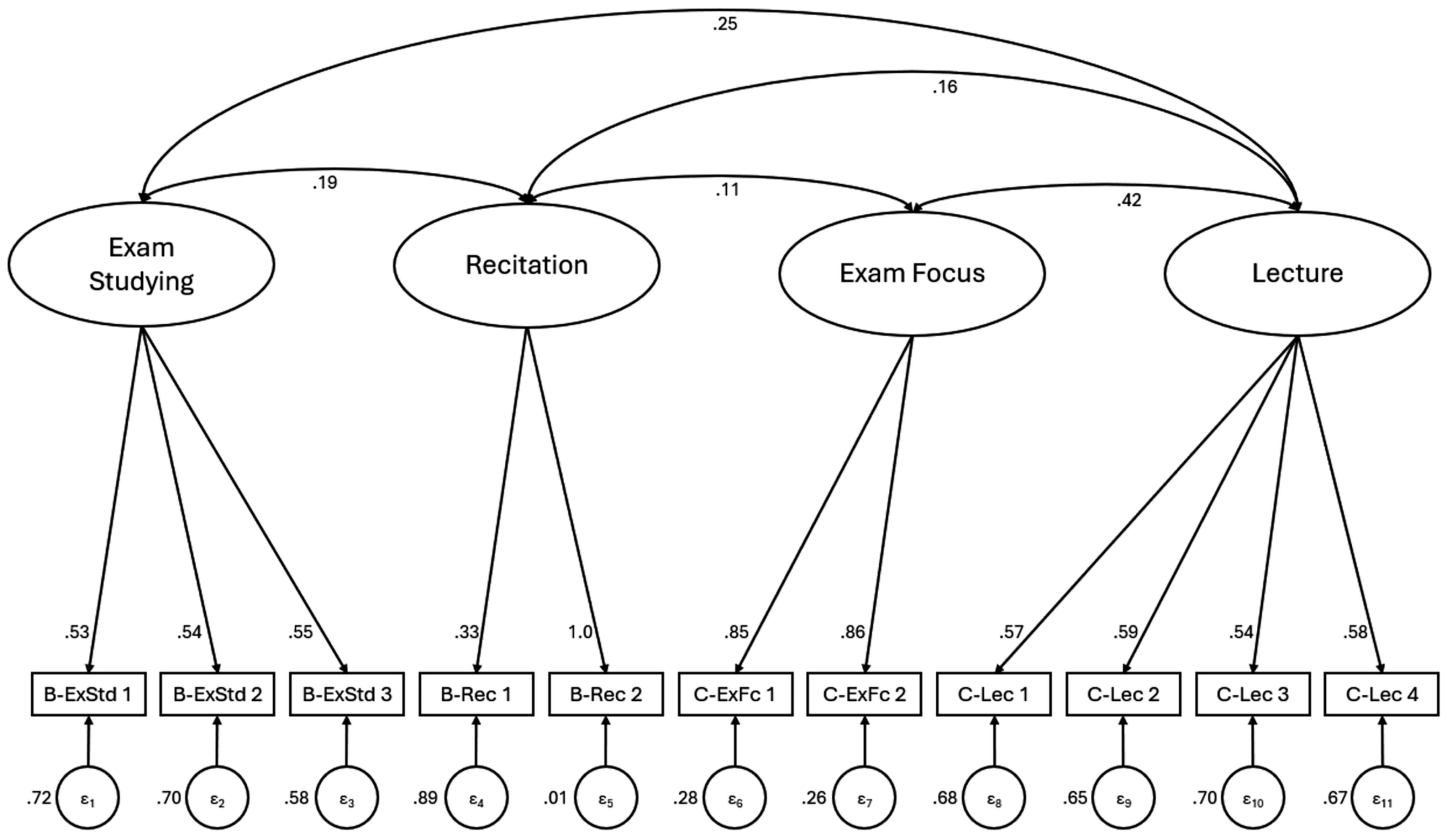
Standardized CFA results of the spaces of engagement hypothesized structure.

Contrarily, the activity space model showed adequate-to-good fit (CFI = 0.94; TLI = 0.92; RMSEA = 0.054, [90% CI = 0.043, 0.064, *p*-RMSEA < 0.05 = 0.275]; SRMR = 0.048; CD = 1.00, Figure 1). The best-fitting model included significant covariance between all latent factors except exam studying and exam focus. This model also had the strongest TLI and RMSEA values (metrics that penalize overfitting). Unlike the behavioral-cognitive model, all standardized factor loadings were statistically significant (Table 2). The much superior fit and strong parsimony-focused metrics indicate this model better explains the observed structure of behavioral-cognitive engagement, and that its increased complexity is justified (Flora and Flake, 2017).

**Table 2:** Standardized path coefficients of the spaces of engagement model (*n* = 772).

Variable path	Coefficient	Bootstrapped standard error	*p*
Exam studying→			
B-exam studying 1	0.53	0.023	0.000
B-exam studying 2	0.54	0.029	0.000
B-exam studying 3	0.65	0.048	0.000
Recitation→			
B-recitation 1	0.33	0.167	0.049
B-recitation 2	1.00	0.074	0.000
Exam focus→			
C-exam focus 1	0.85	0.067	0.000
C-exam focus 2	0.86	0.057	0.000
Lecture→			
C-lecture 1	0.57	0.024	0.000
C-lecture 2	0.59	0.059	0.000
C-lecture 3	0.54	0.027	0.000
C-lecture 4	0.58	0.044	0.000
Covariances			
Lecture ↔ Recitation	0.16	0.041	0.000
Lecture ↔ Exam focus	0.42	0.035	0.000
Lecture ↔ Exam studying	0.25	0.032	0.000
Recitation ↔ Exam focus	0.11	0.048	0.019
Recitation ↔ Exam studying	0.19	0.054	0.001
Error variances			
B-exam studying 1	0.72	0.025	–
B-exam studying 2	0.70	0.031	–
B-exam studying 3	0.58	0.062	–
B-recitation 1	0.89	0.110	–
B-recitation 2	0.01	0.148	–
C-exam focus 1	0.28	0.114	–
C-exam focus 2	0.26	0.099	–
C-lecture 1	0.68	0.027	–
C-lecture 2	0.65	0.069	–
C-lecture 3	0.70	0.030	–
C-lecture 4	0.67	0.050	–

These results indicate a purely behavioral-cognitive model fails to capture the complexity of learning engagement, whereas activity spaces provide a more empirically robust explanation. Findings support a fundamental shift in engagement conceptualization and suggest potential cross-disciplinary generalizability.

## General discussion

### Interpretation

These two studies challenge existing paradigms by demonstrating that, contrary to prevailing theory (Fredricks et al., 2004), behavioral and cognitive engagement may not operate as distinct psychological traits, but rather as emergent properties structured by students’ activity contexts. Addressing our research question, in the examined courses behavioral and cognitive learning engagement’s structure consisted of a consistent core of factors (in-lecture cognition, in-exam cognitive focus, and exam studying behaviors) accompanied by context-dependent factors that emerge when their concomitant activity spaces are available (discussion/recitation behaviors, group assignment behaviors, and group assignment cognition). The spontaneous emergence of this factor structure in EFAs and its fit to the data in the CFA model, as well as the non-emergence of unified behavioral and cognitive factors in the EFA and the severe lack of fit of the same in the CFA suggest the traditional behavioral-cognitive model of learning engagement is insufficient to describe contextualized engagement and should be reconsidered.

The findings call for an ecological perspective on learning engagement, extending beyond classroom dynamics to capture the full academic experience. Our findings indicate engagement is not merely multidimensional as suggested by prior studies (Appleton et al., 2006; Ben-Eliyahu et al., 2018; Zhoc et al., 2019). Rather, it is a partially-context-based dynamic structure with certain features that are stable across different populations, settings, and pedagogical approaches. This finding substantially extends previous work on the situatedness of learning engagement (Altomonte et al., 2016; Lam et al., 2012; Şeker, 2023; Zhao et al., 2021), suggesting potential inseparability between learning engagement and the contexts in which it occurs. Partially addressing calls for the same (see Azevedo, 2015), a more sophisticated and contextually-appropriate understanding of this psychological structure could support more effective interventions and pedagogy, helping address opportunity gaps for minoritized and underprepared students and advancing STEM equity efforts (Beasley and Fischer, 2012; Estrada et al., 2016; Museus et al., 2011; Witherspoon et al., 2019).

### Limitations and future work

Our findings regarding cognitive versus behavioral dominance within engagement spaces may be influenced by survey item selection. The interdisciplinary research team designed items for theoretical and contextual relevance and validated them through extensive cognitive interviews with diverse participants (see Appendix C). However, further research is needed to determine whether alternative item pools would yield different factor structures.

The low inter-factor correlations among engagement spaces (0.06–0.54) raise important questions: Do these factors represent distinct skills, preferences, or both?; How can engagement theory evolve to better explain these observed structures?; and Do differences in factor loadings across courses reflect differences in course structure or student populations?

The engagement scale developed here provides a tool for testing whether specific forms of cognitive and behavioral engagement matter more than overall engagement levels for outcomes like performance, retention, and cognitive development (Schmidt et al., 2018). Analyzing how students engage in different contexts (including those not assessed here, e.g., labs) could help explain learning outcome variability among otherwise similar students and inform targeted interventions to enhance student success (Estrada et al., 2016; Riegle-Crumb et al., 2019).

Future research should explore how engagement patterns evolve across developmental stages, aiding efforts to support learners through key transition points associated with attrition (Akiha et al., 2018). Furthermore, testing how stable factors like in-class cognitive engagement are across different pedagogies, disciplinary groupings (e.g., humanities), and institutions will produce a clearer view of how generalizable the observed factor structure is. As our sample was drawn from selective, research-intensive institutions, it is an open question whether this structure will generalize to courses with high disengagement (e.g., STEM courses for non-majors), less selective institutions, and teaching-focused institutions. Another open question is whether psychological traits (e.g., metacognitive skill, motivation, or anxiety) moderate engagement within different spaces. Finally, linking these engagement structures to downstream outcomes—such as academic persistence, conceptual learning, or identity development—would further clarify their psychological function.

### Implications

Psychometrically, our findings suggest that engagement should not be measured with generic scales divorced from activity context. Instruments must be designed or interpreted with awareness of the environments in which engagement occurs. Practically, this has important implications: given the minimal covariation between engagement spaces, instructors should build multiple pathways to engagement to accommodate diverse learning approaches. Students engaged in one domain may not engage in others, so teaching strategies should broaden engagement opportunities to foster inclusion and persistence in STEM and remove barriers to high-impact spaces of engagement. For example, recent studies report behavioral engagement in discussion/recitation spaces are critical to academic success (McChesney et al., 2025), but these sessions remain poorly attended. Diversifying engagement strategies may particularly benefit marginalized students, who face multiple challenges that could lead to disengagement from STEM (Barbatis, 2010; McGee et al., 2021; Museus et al., 2011). Finally, practitioners might use the factor structure and instrument presented here to precisely evaluate and address spaces of low engagement in their courses, enacting targeted interventions to counteract growing student disengagement (Allison et al., 2024; Newton and Essex, 2024).

## Conclusion

This study challenges prevailing models of learning engagement by demonstrating that behavioral and cognitive engagement are structured not only by psychological domain, but also by the specific learning contexts—or *activity spaces*—in which they occur, revealing that context not only shapes engagement intensity, but dimensionality as well. Across multiple university STEM courses, factor analyses consistently revealed that engagement patterns aligned with discrete educational settings (e.g., lectures, exams, group work), rather than mapping cleanly onto traditional behavioral-cognitive dimensions. These findings support a reconceptualization of engagement as a context-sensitive psychological construct: shaped by how learners interact with the structural, social, and cognitive demands of academic environments. Rather than treating behavioral and cognitive engagement as stable traits or general tendencies, our results suggest they are emergent, partially modular responses to learning settings.

This refined model of engagement has implications for psychological theory, measurement design, and educational practice. Theoretically, it bridges ecological and cognitive perspectives on learning by highlighting how engagement processes are distributed across contexts. Methodologically, it calls for more precise, context-aware instruments to capture the complexity of engagement. Practically, it encourages instructors and institutions to broaden the range of engagement opportunities in their courses, especially those known to influence retention and success among marginalized learners.

By grounding engagement in the affordances of activity spaces, this study advances a more ecologically valid and psychologically meaningful understanding of how students engage with learning—and how that engagement can be measured, supported, and sustained.

## Data Availability

The raw data supporting the conclusions of this article will be made available by the authors, without undue reservation.
