# Supplementary material for: Reconceptualizing learning engagement: evidence for a context-sensitive structure in STEM education
Source: Front Psychol. 2025 Nov 11;16:1649744. doi: 10.3389/fpsyg.2025.1649744 (PMC12643978; doi:10.3389/fpsyg.2025.1649744)
Supplement: Supplementary file 1 [file Supplementary_file_1.docx]

# Online Only Appendix A: Ancillary Tables and Figures

**Table A1** *Demographic Characteristics of Quantitative Samples*

|  |  | Exploratory Study | | | | Confirmatory Study | | |
| --- | --- | --- | --- | --- | --- | --- | --- | --- |
|  |  | Engineering Design  (*n* = 774) | | Genetics  (*n* = 402) | | | Biology  (*n* = 772) | |
| Race | | *n* | % | *n* | % | | *n* | % |
|  | Black/African American | 16 | 2 | 10 | 2 | | 36 | 5 |
|  | American Indian/Alaskan Native | 7 | 1 | 0 | 0 | | 2 | 0 |
|  | Arab, Middle Eastern, or Persian | 17 | 2 | 7 | 2 | | 16 | 2 |
|  | All Asian | 168 | 22 | 132 | 33 | | 166 | 22 |
|  | All Latinx | 72 | 9 | 18 | 4 | | 42 | 5 |
|  | Native Hawaiian or Pacific Islander | 5 | 1 | 0 | 0 | | 3 | 0 |
|  | White/Caucasian | 426 | 55 | 214 | 53 | | 469 | 61 |
|  | Another ethnicity or prefer not to respond | 63 | 8 | 16 | 4 | | 38 | 5 |
| Gender | |  |  |  |  | |  |  |
|  | Man | 395 | 51 | 116 | 29 | | 173 | 22 |
|  | Woman | 211 | 27 | 227 | 56 | | 447 | 58 |
|  | Another gender or prefer not to respond | 168 | 22 | 59 | 15 | | 152 | 20 |

*Note.* Due to rounding, numbers may not add up to 100%.

**Table A2** *Cognitive* *Engagement Scale Items, Sorted by Space of Engagement*

|  | Engineering Design | | Genetics | |
| --- | --- | --- | --- | --- |
| Stem and Item | Mean | SD | Mean | SD |
| *In-Exam Focus* |  |  |  |  |
| I had a hard time paying attention when I took the most recent test ^a^ |  |  |  |  |
| I had difficulty thinking clearly while I took the most recent test ^a^ |  |  |  |  |
| For the **most recent test**, to what extent was it easy to: |  |  |  |  |
| pay attention. ^c^ | 3.29 | 0.63 | 2.67 | 0.74 |
| think clearly. ^c^ | 3.23 | 0.67 | 2.53 | 0.71 |
| *Cognitive Recitation Engagement* |  |  |  |  |
| During a **typical** **[recitation/lab]** in the last few weeks, how often have you: |  |  |  |  |
| noticed your mind wandering ^b^ |  |  |  |  |
| asked clarifying questions about topics that were unclear ^b^ | 2.42 | 0.73 | 2,58 | 0.78 |
| combined different pieces of information from the course in new ways (topics from different weeks, etc.) ^b^ | 2.99 | 0.60 | 2.86 | 0.61 |
| made pictures, diagrams, charts, or other figures to help understand the course content ^b^ | 2.77 | 0.87 | 2.61 | 0.80 |
| *Cognitive Class/Lecture Engagement* |  |  |  |  |
| During a **typical [lecture/class]** in the last few weeks |  |  |  |  |
| My mind wandered ^a^ |  |  |  |  |
| I always summarized new [lecture/class] material in my own words when taking notes ^a^ | 2.29 | 0.80 | 2.78 | 0.73 |
| when I had difficulty understanding [lecture/class] material, I marked it to come back to later^a^ | 2.70 | 0.77 | 3.11 | 0.70 |
| I focused on understanding the diagrams, charts, and figures presented in the [lecture/class] ^a^ | 3.15 | 0.58 | 3.13 | 0.61 |
| In this class I was able to stay focused and on-task____ of the time: |  |  |  |  |
| during [lab/recitation]. ^d^ |  |  | 2.85 | 0.69 |
| during [lecture/class]. ^d^ | 2.85 | 0.66 | 2.66 | 0.67 |
| while working on assignments. ^d^ | 3.10 | 0.59 | 3.01 | 0.67 |
| *Cognitive Group Work Engagement* |  |  |  |  |
| During the latest group assignment: |  |  |  |  |
| I made sure to understand the plan for the project and my role in that plan^a^ | 3.46 | 0.54 |  |  |
| I was able to stay mentally focused while completing **my part of** the project ^a^ | 3.40 | 0.60 |  |  |

*Note*: Response options are indicated as follows: ^a^ Strongly disagree, Disagree, Agree, Strongly agree; ^b^ Never, Rarely, Often, Always; ^c^ Very difficult, Difficult, Easy, Very easy; ^d^ None, Some, Most, All, Not applicable

**Table A3** *Behavioral* *Engagement Scale Items, Sorted by Space of Engagement*

|  |  |  | Engineering Design | | Genetics | |
| --- | --- | --- | --- | --- | --- | --- |
| Stem and Item |  |  | Mean | SD | Mean | SD |
| *Exam Studying* | | |  |  |  |  |
| While studying for the most recent exam or midterm: | | |  |  |  |  |
| other students quizzed me or asked for my help ^a^ | | |  |  |  |  |
| I spent _____ reorganizing my notes so the big ideas were clear ^b^ | | | 2.72 | 1.26 | 5.01 | 1.88 |
| *No stem was used in the following items* | | |  |  |  |  |
| I started studying for the most recent exam____ ^c^ | | | 2.12 | 0.75 | 3.11 | 0.70 |
| I spent __ hours studying alone for the most recent exam. ^d^ | | | 1.18 | 1.29 | 3.13 | 0.61 |
| I spent __ hours actively studying with classmates for the most recent exam. ^d^ | | | 0.41 | 0.82 |  |  |
| I went to the instructor or TA's office hours while studying for the most recent exam __ ^e^ | | |  |  | 2.78 | 0.70 |
| *Behavioral Recitation Engagement* | | |  |  |  |  |
| I have attended _________ of the [recitations/labs] so far ^f^ | | | 3.58 | 0.50 | 3.58 | 0.54 |
| I ________ worked with other students during [recitation/labs] ^g^ | | |  |  |  |  |
| I completed ______ of the activities we were given in [recitation/labs] ^f^ | | | 3.91 | 5.97 | 3.72 | 0.52 |
| *Behavioral Lecture/Class Engagement* | | |  |  |  |  |
| I have attended _____ of the class [lecture/class]s so far ^h^ | | |  |  |  |  |
| I _____ participated in the in-class activities during [lecture/class] (clicker response questions, hand raising, think-pair-share, etc.) ^i^ | | |  |  |  |  |
| *Behavioral Group Work Engagement* | | |  |  |  |  |
| I spent ___ hours with others on my team to complete the group project. ^d^ | | | 7.72 | 12.30 |  |  |
| I spent ___ hours on my own working on the team project. ^d^ | | | 5.44 | 7.58 |  |  |
| *Behavioral Assignment Engagement* | | |  |  |  |  |
| On the most difficult parts of each assignment, I ___^j^ | | |  |  |  |  |

*Note*: Response options are indicated as follows: ^a^ Never, Once, Rarely, Multiple times; ^b^ 0 minutes, 15 minutes, 30 minutes, 1 hour, 2 hours, 3 hours, 4+ hours; ^c^ the day of, the day before, several days before, a week or more before; ^d^ Numerical input ; ^e^ Never, Once, Twice, More than twice; ^f^ None, Some, Most, All, Not applicable; ^g^ Never, Seldom, Often, Always, Not applicable; ^h^ Some, Most, Almost all, All; ^i^ Never, Once, Rarely, Always; ^j^ Quickly took my best guess, Spent some time working on it, Spent much time working on it, Took as much time as needed

**Figure A1**

*Engineering Design Scree Plot of Eigenvalues by Factor Number*


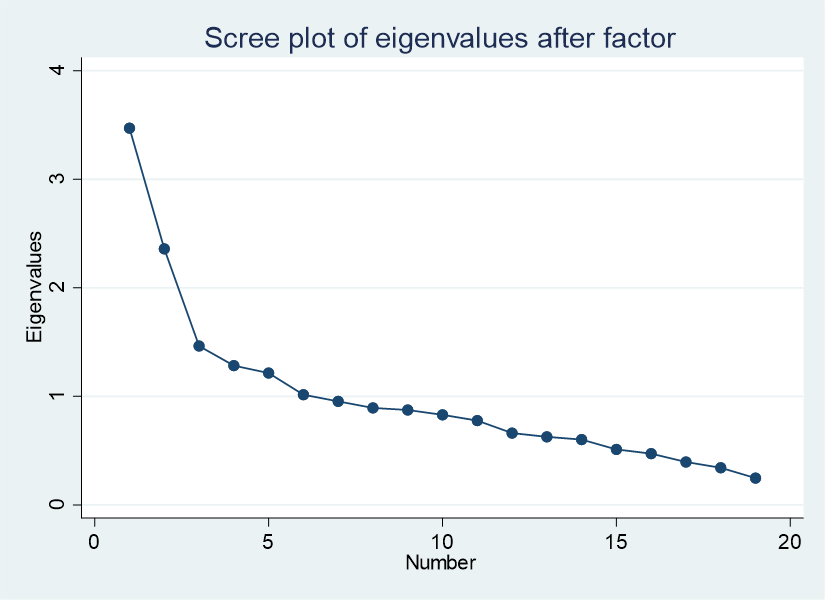


**Figure A2**

*Genetics Scree Plot of Eigenvalues by Factor Number*


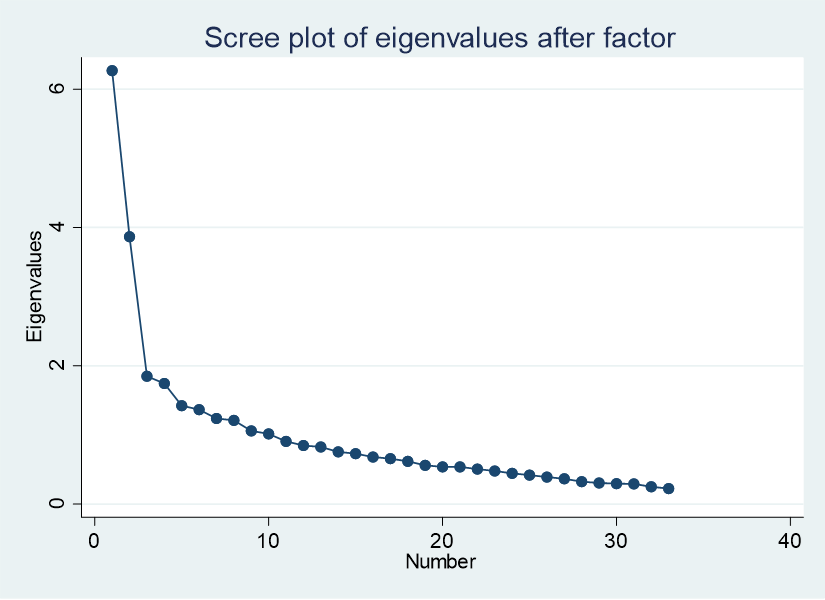


**Table A4** *Cronbach’s Alpha Reliability of Scale Factors*

| Factor | Engineering Design | Genetics |
| --- | --- | --- |
| Exam Focus | .86 | .79 |
| Recitation |  | .61 |
| In Lecture/Class |  | .70 |
| Exam Studying | .74 | .56 |
| Recitation and Lecture | .69 |  |
| Cognitive Group Assignment | .75 |  |
| Time on Group Assignment | .83 |  |

**Table A5**

*Dropped (x) or Loaded (√) Status of* *Cognitive* *Engagement Scale Items, Sorted by Space of Engagement*

| Stem and Item | Engineering Design | Genetics |
| --- | --- | --- |
| *In-Exam Focus* |  |  |
| For the **most recent test**, to what extent was it easy to: |  |  |
| pay attention. ^c^ | √ | √ |
| think clearly. ^c^ | √ | √ |
| *Cognitive Recitation Engagement* |  |  |
| During a **typical** **[recitation/lab]** in the last few weeks, how often have you: |  |  |
| asked clarifying questions about topics that were unclear ^b^ | √ | √ |
| combined different pieces of information from the course in new ways (topics from different weeks, etc.) ^b^ | √ | x |
| made pictures, diagrams, charts, or other figures to help understand the course content ^b^ | √ | √ |
| *Cognitive Class/Lecture Engagement* |  |  |
| During a **typical [lecture/class]** in the last few weeks |  |  |
| I always summarized new [lecture/class] material in my own words when taking notes ^a^ | √ | √ |
| when I had difficulty understanding [lecture/class] material, I marked it to come back to later^a^ | √ | √ |
| I focused on understanding the diagrams, charts, and figures presented in the [lecture/class] ^a^ | √ | √ |
| In this class I was able to stay focused and on-task____ of the time: |  |  |
| during [lab/recitation]. ^d^ |  | x |
| during [lecture/class]. ^d^ | x | x |
| while working on assignments. ^d^ | x | x |
| *Cognitive Group Work Engagement* |  |  |
| During the latest group assignment: |  |  |
| I made sure to understand the plan for the project and my role in that plan^a^ | √ |  |
| I was able to stay mentally focused while completing **my part of** the project ^a^ | √ |  |

*Note*: Response options are indicated as follows: ^a^ Strongly disagree, Disagree, Agree, Strongly agree; ^b^ Never, Rarely, Often, Always; ^c^ Very difficult, Difficult, Easy, Very easy; ^d^ None, Some, Most, All, Not applicable

**Table A6**

*Dropped (x) or Loaded (√) Status of* *Behavioral* *Engagement Scale Items, Sorted by Space of Engagement*

| Stem and Item | Engineering Design | Genetics |
| --- | --- | --- |
| *Exam Studying* |  |  |
| While studying for the most recent exam or midterm: |  |  |
| other students quizzed me or asked for my help ^a^ |  |  |
| I spent _____ reorganizing my notes so the big ideas were clear ^b^ | √ | √ |
| *No stem was used in the following items* |  |  |
| I started studying for the most recent exam____ ^c^ | √ | √ |
| I spent __ hours studying alone for the most recent exam. ^d^ | √ | √ |
| I spent __ hours actively studying with classmates for the most recent exam. ^d^ | x | x |
| I went to the instructor or TA's office hours while studying for the most recent exam __ ^e^ |  | x |
| *Behavioral Recitation Engagement* |  |  |
| I have attended _________ of the [recitations/labs] so far ^f^ |  | √ |
| I ________ worked with other students during [recitation/labs] ^g^ |  | x |
| I completed ______ of the activities we were given in [recitation/labs] ^f^ |  | √ |
| *Behavioral Lecture/Class Engagement* |  |  |
| I have attended _____ of the course [lectures/classes] so far ^h^ |  | x |
| I _____ participated in the in-class activities during [lecture/class] (clicker response questions, hand raising, think-pair-share, etc.) ^i^ | x | x |
| *Behavioral Group Work Engagement* |  |  |
| I spent ___ hours with others on my team to complete the group project. ^d^ | √ | x |
| I spent ___ hours on my own working on the team project. ^d^ | √ | x |
| *Behavioral Assignment Engagement* |  |  |
| On the most difficult parts of each assignment, I ___^j^ |  |  |

*Note*: Response options are indicated as follows: ^a^ Never, Once, Rarely, Multiple times; ^b^ 0 minutes, 15 minutes, 30 minutes, 1 hour, 2 hours, 3 hours, 4+ hours; ^c^ the day of, the day before, several days before, a week or more before; ^d^ Numerical input ; ^e^ Never, Once, Twice, More than twice; ^f^ None, Some, Most, All, Not applicable; ^g^ Never, Seldom, Often, Always, Not applicable; ^h^ Some, Most, Almost all, All; ^i^ Never, Once, Rarely, Always; ^j^ Quickly took my best guess, Spent some time working on it, Spent much time working on it, Took as much time as needed

**Table A7**

*CFA Items and Descriptive Statistics*

| Variable and Item | Mean | SD | Min | Max |
| --- | --- | --- | --- | --- |
| **B-Exam Studying 1** | 5.234 | 1.774 | 1 | 7 |
| While studying for the most recent exam or midterm: I spent __ reorganizing my notes so the big ideas were clear. |  |  |  |  |
| **B-Exam Studying 2** | 3.285 | .629 | 1 | 4 |
| I started studying for the most recent exam __. |  |  |  |  |
| **B-Exam Studying 3*** | 2.704 | 1.117 | 1 | 4 |
| I spent __ hours studying alone for the most recent exam. |  |  |  |  |
| **B-Recitation 1** | .746 | .436 | 0 | 1 |
| I have attended __of the recitations so far. |  |  |  |  |
| **B-Recitation 2** | .786 | .410 | 0 | 1 |
| I completed __ of the activities we were given in recitation. |  |  |  |  |
| **C-Exam Focus 1** | 2.845 | .730 | 1 | 4 |
| For the most recent test, to what extent was it easy to: pay attention? |  |  |  |  |
| **C-Exam Focus 2** | 2.657 | .743 | 1 | 4 |
| For the most recent test, to what extent was it easy to: think clearly? |  |  |  |  |
| **C-Lecture 1** | 3.031 | .619 | 1 | 4 |
| During a typical lecture in the last few weeks, how often have you: combined different pieces of information from the course in new ways (topics from different weeks, etc.) |  |  |  |  |
| **C-Lecture 2** | 2.804 | .748 | 1 | 4 |
| During a typical lecture in the last few weeks, how often have you: made pictures, diagrams, charts, or other figures to help understand the course content |  |  |  |  |
| **C-Lecture 3** | 2.863 | .713 | 1 | 4 |
| During a typical lecture in the last few weeks: I always summarized new lecture material in my own words when taking notes |  |  |  |  |
| **C-Lecture 4** | 3.197 | .556 | 1 | 4 |
| During a typical lecture in the last few weeks: I focused on understanding the diagrams, charts, and figures presented in the lecture |  |  |  |  |

**Table A8**

*Skewness and Kurtosis χ^2^ Test of Univariate Normality of CFA Variables (n = 772)*

| Variable | | Pr(skewness) | Pr(kurtosis) | Adj. χ^2^ | Prob> χ^2^ |
| --- | --- | --- | --- | --- | --- |
| B-Exam Studying 1 | 0.000 | | 0.015 | 54.880 | 0.000 |
| B-Exam Studying 2 | 0.000 | | 0.467 | 22.360 | 0.000 |
| B-Exam Studying 3* | 0.141 | | . | . | . |
| B-Recitation 1 | 0.000 | | 0.000 | 119.940 | 0.000 |
| B-Recitation 2 | 0.000 | | 0.872 | 116.640 | 0.000 |
| C-Exam Focus 1 | 0.001 | | 0.675 | 9.940 | 0.007 |
| C-Exam Focus 2 | 0.955 | | 0.009 | 6.720 | 0.035 |
| C-Lecture 1 | 0.000 | | 0.001 | 22.190 | 0.000 |
| C-Lecture 2 | 0.007 | | 0.192 | 8.590 | 0.014 |
| C-Lecture 3 | 0.039 | | 0.171 | 6.140 | 0.046 |
| C-Lecture 4 | 0.010 | | 0.000 | 23.770 | 0.000 |

*Note: the - in Pr(kurtosis) indicates a very high value that cannot be calculated using Royston's (1991) correction to D’Agostino and colleagues' (1990) normality test. It should be interpreted as indicating non-normality.

**Table A9**

*Shapiro-Wilk Test of Univariate Normality of CFA Variables (n = 772)*

| Variable | W | V | z | Prob>z |
| --- | --- | --- | --- | --- |
| B-Exam Studying 1 | 0.980 | 10.112 | 5.668 | 0.000 |
| B-Exam Studying 2 | 0.984 | 7.870 | 5.054 | 0.000 |
| B-Exam Studying 3 | 0.994 | 2.895 | 2.604 | 0.005 |
| B-Recitation 1 | 0.996 | 1.771 | 1.400 | 0.081 |
| B-Recitation 2 | 0.995 | 2.723 | 2.454 | 0.007 |
| C-Exam Focus 1 | 0.997 | 1.304 | 0.650 | 0.258 |
| C-Exam Focus 2 | 0.999 | 0.614 | -1.194 | 0.884 |
| C-Lecture 1 | 0.992 | 3.795 | 3.268 | 0.001 |
| C-Lecture 2 | 0.998 | 0.950 | -0.125 | 0.550 |
| C-Lecture 3 | 0.998 | 1.245 | 0.536 | 0.296 |
| C-Lecture 4 | 0.981 | 9.322 | 5.469 | 0.000 |


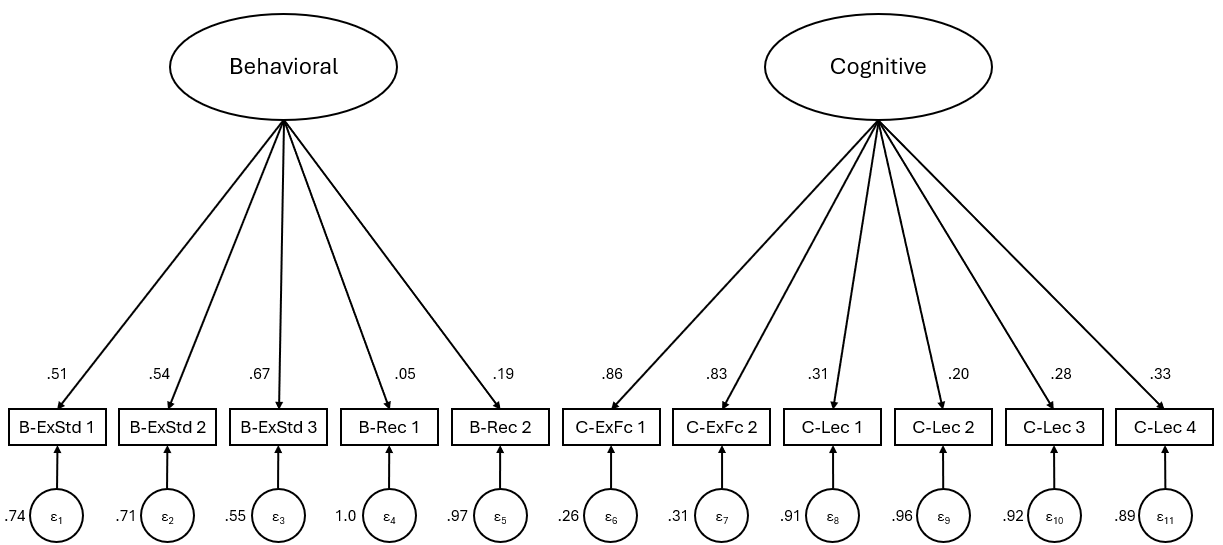


**Figure A3.** *Standardized CFA Results of the Behavioral-Cognitive Hypothesized Structure*

**Table A10**

*Standardized Path Coefficients of the Behavioral-Cognitive Hypothesized Structure (n = 772)*

| Variable Path | | Coefficient | Bootstrapped Standard Error | *p* |
| --- | --- | --- | --- | --- |
| Behavioral→ | |  |  |  |
|  | B-Exam Studying 1 | .51 | .034 | .000 |
|  | B-Exam Studying 2 | .54 | .034 | .000 |
|  | B-Exam Studying 3 | .67 | .054 | .000 |
|  | B-Recitation 1 | .05 | .081 | .517 |
|  | B-Recitation 2 | .19 | .036 | .000 |
| Cognitive→ | |  |  |  |
|  | C-Exam Focus 1 | .86 | .034 | .000 |
|  | C-Exam Focus 2 | .83 | .026 | .000 |
|  | C-Lecture 1 | .31 | .032 | .000 |
|  | C-Lecture 2 | .20 | .052 | .000 |
|  | C-Lecture 3 | .28 | .038 | .000 |
|  | C-Lecture 4 | .33 | .031 | .000 |
| Error variances | |  |  |  |
|  | B-Exam Studying 1 | .74 | .035 | - |
|  | B-Exam Studying 2 | .71 | .037 | - |
|  | B-Exam Studying 3 | .55 | .073 | - |
|  | B-Recitation 1 | 1.00 | .008 | - |
|  | B-Recitation 2 | .97 | .013 | - |
|  | C-Exam Focus 1 | .26 | .059 | - |
|  | C-Exam Focus 2 | .31 | .043 | - |
|  | C-Lecture 1 | .91 | .020 | - |
|  | C-Lecture 2 | .96 | .021 | - |
|  | C-Lecture 3 | .92 | .021 | - |
|  | C-Lecture 4 | .89 | .020 | - |

# Appendix B: Pilot Tests and Replication Study

## Pilot Study

The pilot study, conducted in Spring 2022 at a large, public, research-intensive U.S. West Coast university, aimed to assess if university STEM students distinguished cognitive and behavioral engagement in various specific learning contexts of the course (e.g., in-class versus in-exam). A 100% response rate (*n* = 149) was achieved in a first-year engineering computations course (see Table B1 for demographics). The engagement instrument, based on prior scales [(Ben-Eliyahu et al., 2018)](https://www.zotero.org/google-docs/?8tKMBu), included seven cognitive and three behavioral items rated on a 4-point Likert scale. The instrument and its response characteristics are available in Appendix Table B2. The demographic instrument measured student race/ethnicity and gender at a granular level (e.g., East Asian, Southeast Asian, etc.). For brevity, these categories have been aggregated into broader categories (e.g., Asian) in the demographic summary tables provided in Table B1.

**Table B1** *Demographic Characteristics of Quantitative Samples*

|  | |  | Pilot | | | Replication Study 1 | | | | | Replication Study 2 | | | |
| --- | --- | --- | --- | --- | --- | --- | --- | --- | --- | --- | --- | --- | --- | --- |
|  | |  | Engineering Comp. (*n* = 149) | | Org. Chemistry  (*n* = 198) | | | Economics  (*n* = 324) | | Gen. Chemistry 2 (*n* = 346) | | | Engineering Coding (*n* = 810) | |
|  | |  | *n* | % | *n* | | % | *n* | % | *n* | | % | *n* | % |
| Race | | |  |  |  | |  |  |  |  | |  |  |  |
|  | Black/African American | | 11 | 2 | 9 | | 5 | 28 | 9 | 11 | | 3 | 19 | 2 |
|  | American Indian/Alaskan Native | | 6 | 1 | 0 | | 0 | 2 | 1 | 0 | | 0 | 2 | 0 |
|  | Arab, Middle Eastern, or Persian | | 16 | 3 | 9 | | 5 | 11 | 3 | 0 | | 0 | 24 | 3 |
|  | All Asian | | 130 | 25 | 105 | | 53 | 74 | 23 | 97 | | 29 | 221 | 27 |
|  | All Latinx | | 43 | 8 | 3 | | 2 | 22 | 7 | 20 | | 6 | 72 | 9 |
|  | Native Hawaiian or Pacific Islander | | 3 | 0.6 | 1 | | 1 | 0 | 0 | 0 | | 0 | 2 | 0 |
|  | White/Caucasian | | 464 | 71 | 90 | | 46 | 241 | 74 | 197 | | 54 | 380 | 47 |
|  | Another ethnicity or prefer not to respond | | 2 | 0 | 20 | | 10 | 7 | 2 | 8 | | 2 | 5 | 1 |
| Gender | | |  |  |  | |  |  |  |  | |  |  |  |
|  | Man | | 369 | 71 | 44 | | 23 | 164 | 51 | 61 | | 24 | 513 | 63 |
|  | Woman | | 140 | 27 | 145 | | 74 | 167 | 52 | 184 | | 72 | 270 | 33 |
|  | Another gender or prefer not to respond | | 4 | 1 | 6 | | 3 | 4 | 1 | 7 | | 3 | 26 | 2 |

*Note.* Due to rounding, numbers may not add up to 100%.

**Table B2**

*Pilot Study Engagement Scale*

| Domain and Item | Mean | SD | Cronbach’s ɑ |
| --- | --- | --- | --- |
| Cognitive Exam Engagement |  |  | .90 |
| I had a hard time paying attention when I took the midterm. -R | 2.88 | 0.73 |  |
| I had difficulty thinking clearly while I took the midterm. -R | 2.68 | 0.83 |  |
| My mind wandered when I took the midterm. -R | 2.59 | 0.83 |  |
| I had a hard time organizing my thoughts when I took the midterm. -R | 2.55 | 0.80 |  |
| Class Behavioral Engagement |  |  | .15 |
| I made sure to complete every part of every assignment | 3.16 | 0.58 |  |
| I put a lot of time into studying for exams | 2.45 | 0.76 |  |
| I sometimes had a hard time staying motivated to complete homework -R | 2.22 | 0.69 |  |
| Class Cognitive Engagement |  |  | .42 |
| I regularly spent time thinking about connections between different things we were learning | 2.80 | 0.61 |  |
| I was able to stay focused while doing the assignments | 2.82 | 0.60 |  |
| My mind often wandered to outside topics during class - R | 2.38 | 0.71 |  |

*Note: -*R indicates item was reverse coded

Data were collected online via a Qualtrics survey, with demographics administered at the beginning of the Spring 2022 term and the engagement survey given at the end of that term, prior to the final exam period. Students were given a small number of participation points for beginning the survey. To enhance honest responding, students were reassured that their instructors would not have access to any information they volunteered on the survey, that their responses would be deidentified before analysis, and that their responses would only be reported in the aggregate. In addition to including reverse-coded items to control for acquiescence bias, an item was included in the survey to identify inattentive respondents [(Barge & Gehlbach, 2012)](https://www.zotero.org/google-docs/?jYlEP9) that read “If you are reading this question, check ‘Strongly disagree.’”

Confirmatory factor analysis (CFA) in Stata v.17 using the *sem* package and maximum likelihood estimation indicated that a two-factor model did not fit the data, but an alternative model (presented in Figure B1 and Table B3) incorporating "spaces of engagement" showed better fit (Brown, 2015; Flora et al., 2012). In particular, there were separate factors for in-class and in-exam cognitive engagement. However, the instrument was not optimized to capture space-specific factors within cognitive and behavioral engagement, and thus a revised instrument and corresponding data collection was needed.

**Figure B1**

*Pilot Study Instrument Alternative Confirmatory Factor Model with Exam Activities as a Separate Space of Engagement*


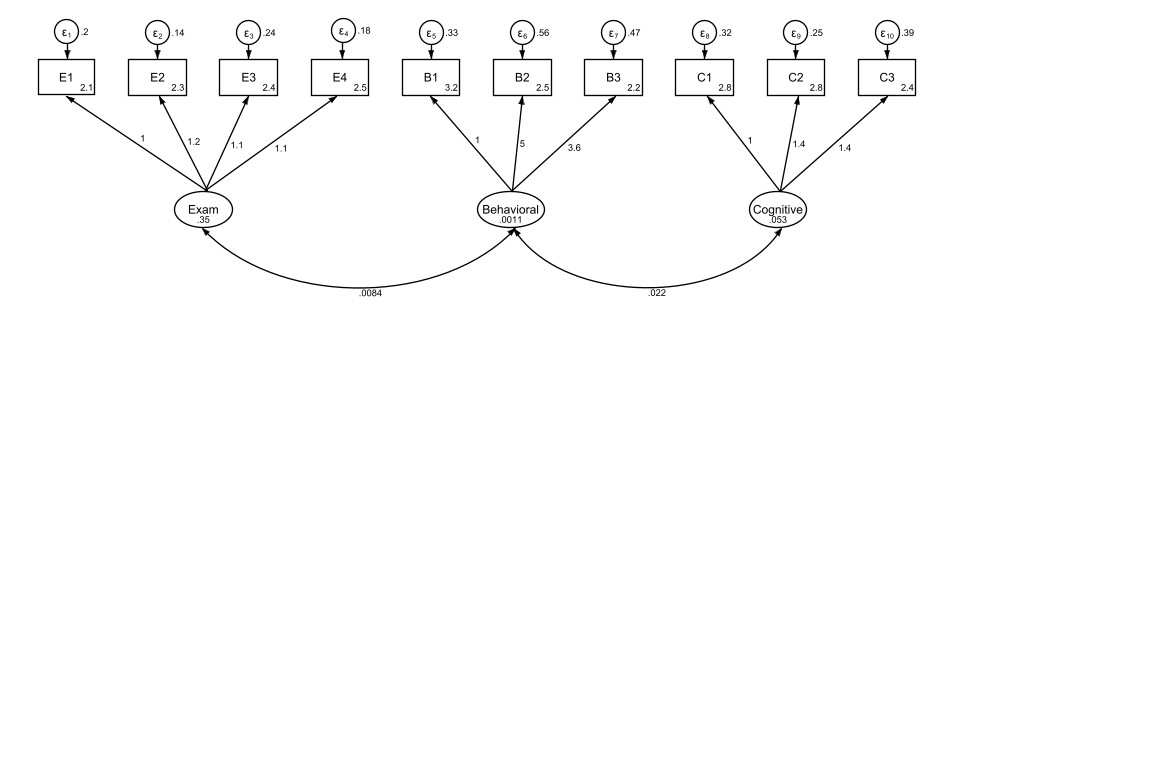
 **Table B3**

*Fit Indices for Pilot Study Spaces of Engagement Model*

| Index | Estimate |
| --- | --- |
| CFI | .89 |
| TLI | .85 |
| RMSEA | .108 |
| SRMR | .104 |

## Replication Study 1

In Fall 2022, a revised and expanded engagement scale was tested at a large, research-intensive, Mid-Atlantic university in organic chemistry, macroeconomics, and microeconomics courses (see Table B1 for demographics). There was a 69% response rate in both courses with *n* = 198 remaining for chemistry and *n* = 324 remaining for economics after cleaning and discarding non-valid responses as indicated by the attention check item. The revised instrument included 10 behavioral and 10 cognitive items with varied response options to reduce acquiescence and inattentive bias [(Barge & Gehlbach, 2012)](https://www.zotero.org/google-docs/?m8zj9h). Exploratory factor analysis with maximum likelihood estimation and promax factor rotation in Stata v.17 revealed a four-factor structure based on factor eigenvalues, scree plots (presented in Appendix Figures B2 and B3), factor loading coherence, item sufficiency, absence of cross-loading items, and theoretical interpretability. Factor loadings are available in Table B4. Factor contents were similar across contexts, indicating structural consistency. The study supported separation by behavioral and cognitive aspects and spaces of engagement; however, reverse-coded items formed a confounded methods factor. Consequently, all reverse-coded items were replaced with positively worded items before testing the scale in larger samples.

**Figure B2**

*Organic Chemistry Scree Plot of Eigenvalues by Factor Number*


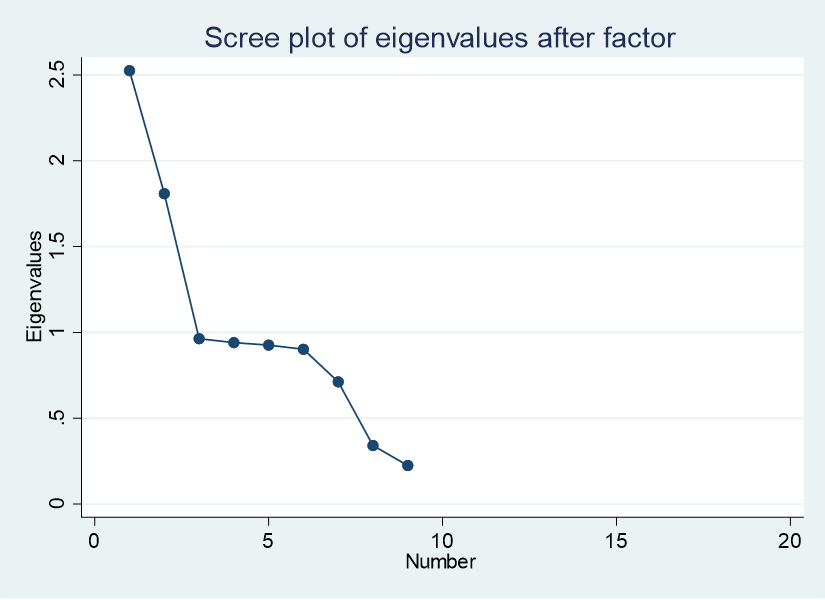


**Figure B3**

*Economics Scree Plot of Eigenvalues by Factor Number*


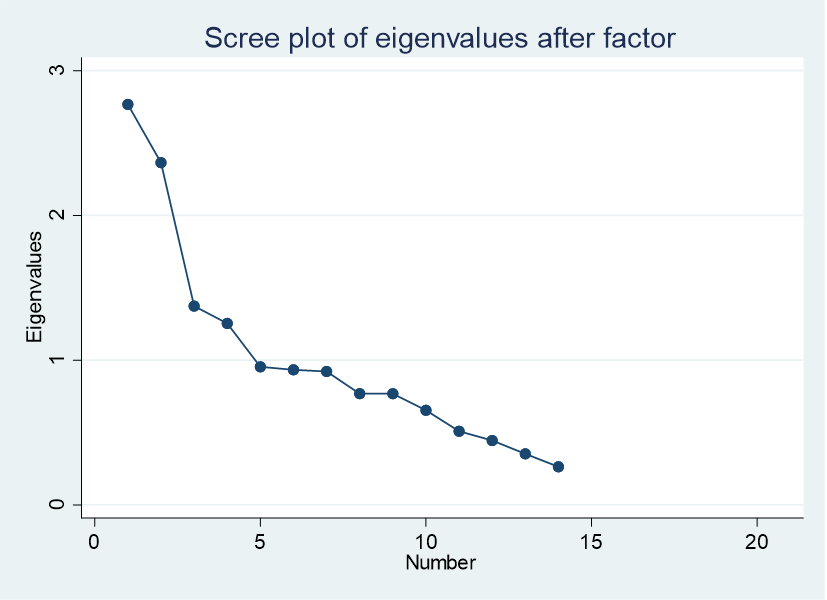


**Table B4**

*Replication Study 1 Rotated Factor Loadings Within Each Course, Organized by Location and Behavioral (black) vs. Cognitive (blue) Focus.*

| **Item** | **Organic Chemistry (*n* = 198)** | | | |  | **Economics (*n* = 324)** | | | |
| --- | --- | --- | --- | --- | --- | --- | --- | --- | --- |
|  | Factor 1 Exam | Factor 2  Recitation | Factor 3  Cognitive strategies | Factor 4  Studying |  | Factor 1  Exam | Factor 2  Recitation | Factor 3  Cognitive strategies | Factor 4  Studying |
| *C1: I had a hard time paying attention when I took the most recent test | **.97** |  |  |  |  | **.81** |  |  |  |
| *C2: I had difficulty thinking clearly while I took the most recent test | **.86** |  |  |  |  | **1.01** |  |  |  |
| B5: I completed __ of the activities we were given in recitation |  | **.64** |  |  |  |  | .47 |  |  |
| B3: I have attended __ of the recitations so far |  |  |  |  |  |  | **.91** |  |  |
| C4: During a typical recitation, how often have you asked clarifying questions about topics that were unclear |  | .48 |  |  |  |  |  |  |  |
| C5: During a typical recitation, how often have you combined different pieces of information from the course in new ways (topics from different weeks, etc.) |  | .44 |  |  |  |  |  | **.61** |  |
| C6: During a typical recitation, how often have you made pictures, diagrams, charts, or other figures to help understand the course content |  |  | .43 |  |  |  |  | .43 |  |
| *C3: During a typical recitation, how often have you noticed your mind wandering | .50 |  |  |  |  |  |  | .44 |  |
| C10: During a typical lecture, I focused on understanding the diagrams, charts, and figures presented in the lecture |  |  | **.82** |  |  |  |  | .43 |  |
| *C7: During a typical lecture, my mind wandered | .44 |  |  |  |  |  |  | .43 |  |
| C8: I always summarized new lecture material in my own words when taking notes |  |  |  |  |  |  |  | .56 |  |
| C9: When I had difficulty understanding lecture material, I marked it to come back to later |  |  | .52 |  |  |  |  |  |  |
| B2: While studying for the most recent exam or midterm I spent __ reorganizing my notes so the big ideas were clear |  |  |  |  |  |  |  |  | **.73** |
| B9: I started studying for the most recent exam __ |  |  |  |  |  |  |  |  | **.66** |
| B10: I spent __ hours studying alone for the most recent exam. |  |  |  | **.69** |  |  |  |  | **.80** |
| B11: I spent __ hours actively studying with classmates for the most recent exam. |  |  |  | **.78** |  |  |  |  | **.61** |
| B8: I went to the instructor or TA's office hours while studying for the most recent exam __ |  | .55 |  |  |  |  |  |  |  |

*Note.* Item loadings < .40 are not shown. * = reverse-coded item. Gray cells indicated expected loadings based upon factor and item content. Red values indicate significant loading on unexpected factors. The strongest loadings (>.6) are bolded. The Kaiser-Meyer-Olkin (KMO) test of sampling adequacy was .76 for chemistry and .67 for economics.

# Replication Study 2

After all reverse-coded items were replaced with positively worded items, some additional items were added where scales were very short, and items corresponding to new spaces of engagement were added (e.g., engagement in group assignments). Items when deployed in the engineering course were also customized to the details of engineering (e.g., adding items that mention group projects). The revised scale ensured there were multiple behavioral and multiple cognitive items associated with exams, with lecture/class, and with group assignments to be sure there was an opportunity to test whether domain and place each contributed to separate factors. Subsequent to these revisions, a series of cognitive interviews were conducted with STEM students from three different disciplines to ensure the content validity of the revised scale and to refine items and response options (see Appendix C).

## Method

### Participants

The revised scale was trialed at a large, research-intensive, urban Mid-Atlantic U.S. institution in an undergraduate general chemistry 2 course (2 sections), and at a large, research-intensive suburban Midwestern U.S. institution in an undergraduate first-year engineering coding and data analysis course (13 sections). General chemistry 2 focused on conveying an understanding of the kinetics, dynamics, and other fundamental properties of matter, along with applications in industrial and environmental chemistry. This course was accompanied by a laboratory component. Engineering coding focused on using MATLAB and other modern engineering programs to model engineering problems and systems and their potential solutions. Both courses are traditionally taken in the first year of study. The overall response rates were 71% (*n* = 381) and 64% (*n* = 947), respectively. After removing invalid data, 346 responses remained for chemistry and 887 for engineering coding, for sample response rates of 65% and 60%, respectively. The samples’ demographic characteristics were diverse by way of gender (67% women and 2% agender or another gender in chemistry and 33% and 1% respectively in engineering) and by way of race/ethnicity (40% students of color in chemistry and 33% in engineering). Of these, a preponderance (19%) identified as Indian, Pakistani, or Bangladeshi in chemistry. In the engineering coding context 10% identified similarly with a further 11% identifying as East Asian. These demographic characteristics are largely representative of national undergraduate chemistry and engineering enrollments (National Center for Education Statistics, 2023). Demographic characteristics of these samples are reported in Table B1.

### Measures

*Engagement.* Items that did not load in particular pilot/replication study courses were kept because of the observed variation in which items loaded in each factor across courses: it was possible that these items would be important in other course contexts. Three behavioral and six cognitive items were added to the scale to cover the nature of learning activities in the replication study courses. All scale items used a 4-point Likert-type response scale with the exception of two behavioral items that required numerical input. An example of an added behavioral item is “I spent ___ hours with others on my team to complete the group project,” while an example added cognitive item is “During the latest group assignment, I made sure to understand the plan for the project and my role in that plan.” Faculty instructors for each course were consulted regarding class structure (e.g., a single class or a combination of lecture and recitation or lecture and lab) and the suitability of each item to the course context. Response options remained diverse (e.g., including a variety of agreement and frequency scales), and a question to detect inattentive responses was again included to ensure high-quality responses [(Barge & Gehlbach, 2012)](https://www.zotero.org/google-docs/?1kBxin). The instrument and its response characteristics are available in Tables B5 and B6.

**Table B5** *Cognitive* *Engagement Scale Items, Sorted by Space of Engagement*

|  | Replication 1 | | | | Replication 2 | | | | Exploratory Study | | | |
| --- | --- | --- | --- | --- | --- | --- | --- | --- | --- | --- | --- | --- |
|  | Organic Chemistry | | Economics | | General Chemistry 2 | | Engineering Coding | | Engineering Design | | Genetics | |
| Stem and Item | Mean | SD | Mean | SD | Mean | SD | Mean | SD | Mean | SD | Mean | SD |
| *In-Exam Focus* |  |  |  |  |  |  |  |  |  |  |  |  |
| I had a hard time paying attention when I took the most recent test ^a^ | 2.69 | 0.67 | 2.93 | 0.58 |  |  |  |  |  |  |  |  |
| I had difficulty thinking clearly while I took the most recent test ^a^ | 2.52 | 0.72 | 2.93 | 0.60 |  |  |  |  |  |  |  |  |
| For the **most recent test**, to what extent was it easy to: |  |  |  |  |  |  |  |  |  |  |  |  |
| pay attention. ^c^ |  |  |  |  | 2.91 | 0.73 | 3.02 | 0.70 | 3.29 | 0.63 | 2.67 | 0.74 |
| think clearly. ^c^ |  |  |  |  | 2.65 | 0.79 | 3.06 | 0.64 | 3.23 | 0.67 | 2.53 | 0.71 |
| *Cognitive Recitation Engagement* |  |  |  |  |  |  |  |  |  |  |  |  |
| During a **typical** **[recitation/lab]** in the last few weeks, how often have you: |  |  |  |  |  |  |  |  |  |  |  |  |
| noticed your mind wandering ^b^ | 2.27 | 0.69 | 2.47 | 0.72 |  |  |  |  |  |  |  |  |
| asked clarifying questions about topics that were unclear ^b^ | 2.22 | 0.82 | 2.24 | 0.71 | 2.43 | 0.74 | 2.55 | 0.78 | 2.42 | 0.73 | 2,58 | 0.78 |
| combined different pieces of information from the course in new ways (topics from different weeks, etc.) ^b^ | 2.59 | 0.73 | 2.60 | 0.68 | 2.80 | 0.69 | 3.13 | 0.65 | 2.99 | 0.60 | 2.86 | 0.61 |
| made pictures, diagrams, charts, or other figures to help understand the course content ^b^ | 3.03 | 0.70 | 3.17 | 0.69 | 2.42 | 0.83 | 2.60 | 0.91 | 2.77 | 0.87 | 2.61 | 0.80 |
| *Cognitive Class/Lecture Engagement* |  |  |  |  |  |  |  |  |  |  |  |  |
| During a **typical [lecture/class]** in the last few weeks |  |  |  |  |  |  |  |  |  |  |  |  |
| My mind wandered ^a^ | 1.99 | 0.69 | 2.30 | 0.77 |  |  |  |  |  |  |  |  |
| I always summarized new [lecture/class] material in my own words when taking notes ^a^ | 2.66 | 0.70 | 2.79 | 0.68 | 2.68 | 0.70 | 2.19 | 0.81 | 2.29 | 0.80 | 2.78 | 0.73 |
| when I had difficulty understanding [lecture/class] material, I marked it to come back to later^a^ | 3.10 | 0.57 | 2.72 | 0.65 | 3.01 | 0.53 | 2.57 | 0.77 | 2.70 | 0.77 | 3.11 | 0.70 |
| I focused on understanding the diagrams, charts, and figures presented in the [lecture/class] ^a^ | 3.07 | 0.54 | 3.29 | 0.50 | 3.12 | 0.55 | 3.06 | 0.65 | 3.15 | 0.58 | 3.13 | 0.61 |
| In this class I was able to stay focused and on-task____ of the time: |  |  |  |  |  |  |  |  |  |  |  |  |
| during [lab/recitation]. ^d^ |  |  |  |  | 3.08 | 0.66 |  |  |  |  | 2.85 | 0.69 |
| during [lecture/class]. ^d^ |  |  |  |  | 2.85 | 0.71 | 2.67 | 0.67 | 2.85 | 0.66 | 2.66 | 0.67 |
| while working on assignments. ^d^ |  |  |  |  | 2.99 | 0.61 | 3.01 | 0.61 | 3.10 | 0.59 | 3.01 | 0.67 |
| *Cognitive Group Work Engagement* |  |  |  |  |  |  |  |  |  |  |  |  |
| During the latest group assignment: |  |  |  |  |  |  |  |  |  |  |  |  |
| I made sure to understand the plan for the project and my role in that plan^a^ |  |  |  |  | 3.07 | 0.53 | 3.61 | 0.56 | 3.46 | 0.54 |  |  |
| I was able to stay mentally focused while completing **my part of** the project ^a^ |  |  |  |  | 3.12 | 0.55 | 3.49 | 0.65 | 3.40 | 0.60 |  |  |

*Note*: Response options are indicated as follows: ^a^ Strongly disagree, Disagree, Agree, Strongly agree; ^b^ Never, Rarely, Often, Always; ^c^ Very difficult, Difficult, Easy, Very easy; ^d^ None, Some, Most, All, Not applicable

**Table B6** *Behavioral* *Engagement Scale Items, Sorted by Space of Engagement*

|  |  |  | Replication 1 | | | | Replication 2 | | | | Exploratory Study | | | |
| --- | --- | --- | --- | --- | --- | --- | --- | --- | --- | --- | --- | --- | --- | --- |
|  |  |  | Organic Chemistry | | Economics | | General Chemistry 2 | | Engineering Coding | | Engineering Design | | Genetics | |
| Stem and Item |  |  | Mean | SD | Mean | SD | Mean | SD | Mean | SD | Mean | SD | Mean | SD |
| *Exam Studying* | | |  |  |  |  |  |  |  |  |  |  |  |  |
| While studying for the most recent exam or midterm: | | |  |  |  |  |  |  |  |  |  |  |  |  |
| other students quizzed me or asked for my help ^a^ | | | 2.80 | 1.20 | 1.60 | 0.99 | 2.72 | 1.07 | 2.41 | 1.15 |  |  |  |  |
| I spent _____ reorganizing my notes so the big ideas were clear ^b^ | | | 4.45 | 2.10 | 2.45 | 1.68 | 4.94 | 1.89 | 2.52 | 1.39 | 2.72 | 1.26 | 5.01 | 1.88 |
| *No stem was used in the following items* | | |  |  |  |  |  |  |  |  |  |  |  |  |
| I started studying for the most recent exam____ ^c^ | | | 3.71 | 0.45 | 2.53 | 0.68 | 3.34 | 0.58 | 1.86 | 0.91 | 2.12 | 0.75 | 3.11 | 0.70 |
| I spent __ hours studying alone for the most recent exam. ^d^ | | | 9.76 | 8.21 | 2.08 | 2.37 | 8.26 | 6.82 | 1.36 | 2.25 | 1.18 | 1.29 | 3.13 | 0.61 |
| I spent __ hours actively studying with classmates for the most recent exam. ^d^ | | | 4.34 | 4.61 | 0.48 | 0.83 | 2.15 | 2.77 | 0.60 | 1.44 | 0.41 | 0.82 |  |  |
| I went to the instructor or TA's office hours while studying for the most recent exam __ ^e^ | | | 2.02 | 1.15 | 1.11 | 0.37 | 1.77 | 1.03 | 1.29 | 0.73 |  |  | 2.78 | 0.70 |
| *Behavioral Recitation Engagement* | | |  |  |  |  |  |  |  |  |  |  |  |  |
| I have attended _________ of the [recitations/labs] so far ^f^ | | | 3.80 | 0.52 | 3.84 | 0.42 | 3.93 | 0.26 |  |  | 3.58 | 0.50 | 3.58 | 0.54 |
| I ________ worked with other students during [recitation/labs] ^g^ | | | 3.25 | 0.83 | 3.45 | 0.68 | 3.08 | 0.75 | 3.10 | 0.66 |  |  |  |  |
| I completed ______ of the activities we were given in [recitation/labs] ^f^ | | | 3.39 | 0.74 | 3.87 | 0.37 | 3.64 | 0.59 |  |  | 3.91 | 5.97 | 3.72 | 0.52 |
| *Behavioral Lecture/Class Engagement* | | |  |  |  |  |  |  |  |  |  |  |  |  |
| I have attended _____ of the class [lecture/class]s so far ^h^ | | | 3.72 | 0.59 | 3.71 | 0.60 | 3.25 | 0.79 | 3.39 | 0.63 |  |  |  |  |
| I _____ participated in the in-class activities during [lecture/class] (clicker response questions, hand raising, think-pair-share, etc.) ^i^ | | | 2.61 | 1.24 | 2.61 | 1.22 | 2.80 | 1.12 | 3.27 | 0.78 |  |  |  |  |
| *Behavioral Group Work Engagement* | | |  |  |  |  |  |  |  |  |  |  |  |  |
| I spent ___ hours with others on my team to complete the group project. ^d^ | | |  |  |  |  | 0.41 | 1.05 | 4.58 | 3.68 | 7.72 | 12.30 |  |  |
| I spent ___ hours on my own working on the team project. ^d^ | | |  |  |  |  | 0.59 | 1.59 | 2.96 | 2.51 | 5.44 | 7.58 |  |  |
| *Behavioral Assignment Engagement* | | |  |  |  |  |  |  |  |  |  |  |  |  |
| On the most difficult parts of each assignment, I ___^j^ | | |  |  |  |  | 2.99 | 0.82 | 3.39 | 0.73 |  |  |  |  |

*Note*: Response options are indicated as follows: ^a^ Never, Once, Rarely, Multiple times; ^b^ 0 minutes, 15 minutes, 30 minutes, 1 hour, 2 hours, 3 hours, 4+ hours; ^c^ the day of, the day before, several days before, a week or more before; ^d^ Numerical input ; ^e^ Never, Once, Twice, More than twice; ^f^ None, Some, Most, All, Not applicable; ^g^ Never, Seldom, Often, Always, Not applicable; ^h^ Some, Most, Almost all, All; ^i^ Never, Once, Rarely, Always; ^j^ Quickly took my best guess, Spent some time working on it, Spent much time working on it, Took as much time as needed

*Demographics*. The same demographic indices were used as in the pilot and replication studies.

### Procedure

Demographic and engagement data were again gathered via online Qualtrics survey, this time administered at the beginning and end of the Spring 2023 term. All communications and incentive structures were identical to those used in the pilot and replication studies.

### Analysis

The data were examined to ensure they met the assumptions of exploratory factor analysis. The chemistry data exhibited missingness-at-random for all items with the exception of 46 missing responses for the item assessing how often students participated in in-class activities during lecture (e.g., clicker response questions). Because “Never” was a response option for this item, and due to the anomalous number of missing responses, no logic-based imputations were made to the dataset. Instead, the factor analysis utilized full-information maximum likelihood estimation that employed empirical imputation. In the engineering coding sample, data for all items were determined to be missing-at-random apart from responses to “I started studying for the most recent exam__”, which was systematically missing 124 data points. As not studying for the exam at all was not a response option, the unusual missing data pattern was assumed to derive from non-studying participants. Therefore, the missing data was replaced with the lowest available response option (studying the day of). Open numerical response items were tetrachotimized by equal numbers of observations, except for two items, “I spent __ hours with others on my team to complete the group project” and “I spent __hours on my own working on the team project.” Over 79% of respondents input zero hours for both items, indicating they did not participate in any group projects for the course, while the remaining 21% varied broadly in time spent on group projects, likely due to heterogeneity in course assignment practices. These items were tetrachotimized with zero hours indicating the lowest quantile and the remaining quantiles divided into equal numbers of observations. Values greater than three standard deviations from the mean were Winsorized.

Distributional assumptions were investigated via scatter plot matrix and descriptive statistics. The skewness and kurtosis values indicated non-normality for some items; however, assumptions of linearity and unimodality were not violated. Maximum likelihood with missing values estimation was used and factor rotation was conducted with the oblique promax method in Stata v. 17.

## Results

The Kaiser-Meyer-Olkin test for sampling adequacy for the chemistry and engineering coding samples were .76, and .81, respectively, indicating appropriateness for factor analysis [(Flora & Flake, 2017)](https://www.zotero.org/google-docs/?BcdU3e). The optimal number of factors was decided based upon the following criteria: number of factor eigenvalues larger than 1, visual interpretation of the scree plots, strength and coherence of the rotated factor loadings, sufficiency of items per factor extracted, absence of cross-loading items, minimization of items that did not load above .4 on any factor, and the theoretical interpretability of the results. These criteria suggested a five-factor solution for chemistry, and a four-factor solution for engineering coding. The first four factors were essentially the same across courses while the chemistry course added a fifth factor: time spent on the group assignment. Maximum interfactor correlations were .56 in the general chemistry 2 sample and .49 in the engineering coding sample. Scree plots are presented in Figures B4-B5, and rotated factor loadings are reported in Table B7.

**Figure B4**

*General Chemistry 2 Scree Plot of Eigenvalues by Factor Number*


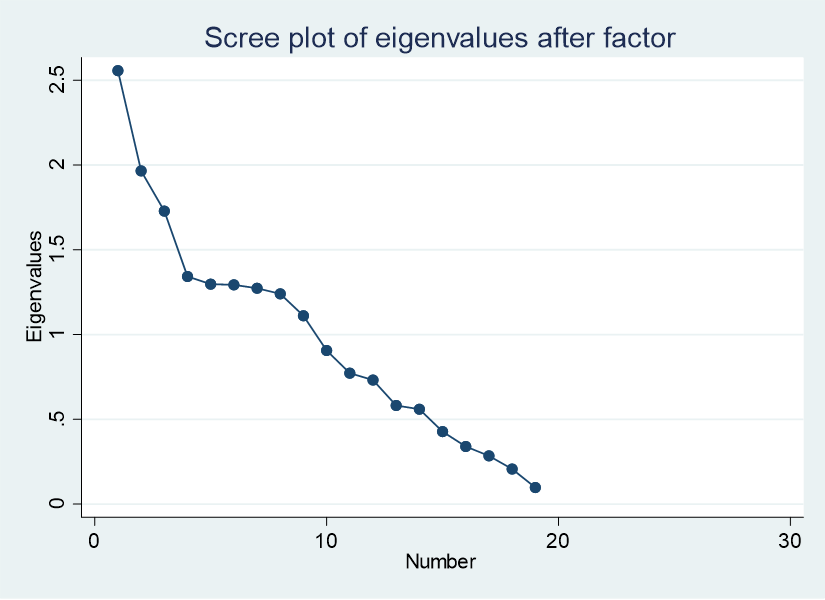


**Figure B5**

*Engineering Coding Scree Plot of Eigenvalues by Factor Number*


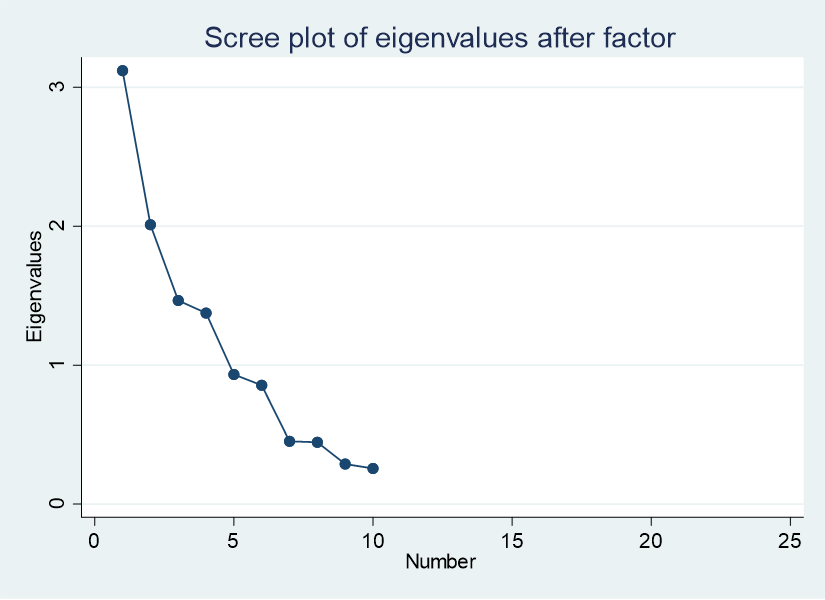


**Table B7**

*Replication Rotated Factor Loadings Within Each Course, Organized by Location and Behavioral(black) vs. Cognitive(blue) Focus.*

| **Item** | **General Chemistry 2 (*n* = 346)** | | | | |  | **Engineering Coding (*n* = 887)** | | | |
| --- | --- | --- | --- | --- | --- | --- | --- | --- | --- | --- |
|  | Factor 1 In-Exam Focus | Factor 2 Pre-Exam Studying | Factor 3 Recitation & Lecture | Factor 4 Group Assign. | Factor 5  Group Time |  | Factor 1 In-Exam Focus | Factor 2  Pre-Exam Studying | Factor 3 Recitation & Lecture | Factor 4 Group Assign. |
| C14: For the most recent test, it was easy to pay attention. | **.80** |  |  |  |  |  | **.91** |  |  |  |
| C15: For the most recent test, it was easy to think clearly. | **.86** |  |  |  |  |  | **.79** |  |  |  |
| B2: While studying for the most recent exam or midterm I spent __ reorganizing my notes so the big ideas were clear |  | .47 |  |  |  |  |  | **.66** |  |  |
| B9: I started studying for the most recent exam [time categories] |  | **.65** |  |  |  |  |  | **.84** |  |  |
| B10: I spent __ hours studying alone for the most recent exam. |  | **.74** |  |  |  |  |  | **.87** |  |  |
| B11: I spent __ hours actively studying with classmates for the most recent exam. |  |  |  |  |  |  |  | .49 |  |  |
| C4: During class, I asked clarifying questions about topics that were unclear |  |  | .42 |  |  |  |  |  | .46 |  |
| C5: During class, I combined different pieces of information from the course in new ways (topics from different weeks, etc.) |  |  |  |  |  |  |  |  | .45 |  |
| C6: During class, I made pictures, diagrams, charts, or other figures to help understand the course content |  |  | .44 |  |  |  |  |  | .48 |  |
| C8: I always summarized new [**class**/**lecture**] material in my own words when taking notes |  |  | .44 |  |  |  |  |  | .52 |  |
| C9: When I had difficulty understanding [**class**/**lecture**] material, I marked it to come back to later |  |  |  |  |  |  |  |  | **.65** |  |
| C10: I focused on understanding the diagrams, charts, and figures presented in the [**class**/**lecture**] |  |  | .47 |  |  |  |  |  | .56 |  |
| C16: I was able to stay focused and on-task__ of the time during recitation. |  |  | **.78** |  |  |  |  |  | skipped |  |
| C17: I was able to stay focused and on-task during class __ of the time. |  |  | **.62** |  |  |  |  |  |  |  |
| C18: I was able to stay focused and on-task__ of the time while working on assignments. |  |  | **.69** |  |  |  |  |  |  |  |
| B5: I completed __ of the activities we were given in [**recitation**/**class**] |  |  | .52 |  |  |  |  |  |  |  |
| C11: During the latest group assignment, I made sure to understand the plan for the project and my role in that plan |  |  |  | **.91** |  |  |  |  |  | **.81** |
| C12: I was able to stay mentally focused while completing my part of the project |  |  |  | **.92** |  |  |  |  |  | **.80** |
| B12: I spent ___ hours with others on my team to complete the group project |  |  |  |  | **.80** |  |  |  |  |  |
| B13: I spent ___ hours on my own working on the team project |  |  |  |  | **1.0** |  |  |  |  |  |
| Average Variance Extracted | .69 | .40 | .32 | .84 | .82 |  | .73 | .53 | .28 | .65 |

*Note.* Item loadings < .40 are not shown. Gray cells indicated expected loadings based upon factor and item content. Bolded text with a / reflect minor wording variation between in each discipline. The strongest loadings (>.6) are bolded.

As in the prior studies, spaces of engagement emerged alongside an almost complete behavioral-cognitive split in both disciplines. The distinction between activity spaces was particularly robust: when a two-factor solution was investigated, the behavioral-cognitive split failed to emerge in either disciplinary context (i.e., only spaces of engagement emerged as factors). The specific space-based factors that emerged were generally identical across the two disciplines: in-exam focus, pre-exam studying, in-class/recitation engagement, group-assignment engagement (see Tables B8 and B9). Two of these factors were entirely behavioral and two of the factors were entirely cognitive (except for a low-load behavioral item, loading in only one discipline). The additional fifth factor in the chemistry context was a behavioral factor also associated with group work, focused on time rather than cognitive engagement. The specific items that loaded were generally shared for three of the four common factors. However, for the classroom-lecture cognitive engagement factor, there was substantial variation in which items loaded at all and which items were the strongly loading items, likely reflecting the differing demands of the courses (e.g., which kinds of cognitive activities were commonly useful). Finally, though cross-loading items were not present in the factorial structure, several items failed to load on any factor in either context.

**Table B8**

*Dropped (x) or Loaded (√) Status of* *Cognitive* *Engagement Scale Items, Sorted by Space of Engagement*

| Stem and Item | General Chemistry 2 | Engineering Coding | Engineering Design | Genetics |
| --- | --- | --- | --- | --- |
| *In-Exam Focus* |  |  |  |  |
| For the **most recent test**, to what extent was it easy to: |  |  |  |  |
| pay attention. ^c^ | √ | √ | √ | √ |
| think clearly. ^c^ | √ | √ | √ | √ |
| *Cognitive Recitation Engagement* |  |  |  |  |
| During a **typical** **[recitation/lab]** in the last few weeks, how often have you: |  |  |  |  |
| asked clarifying questions about topics that were unclear ^b^ | √ | √ | √ | √ |
| combined different pieces of information from the course in new ways (topics from different weeks, etc.) ^b^ | x | √ | √ | x |
| made pictures, diagrams, charts, or other figures to help understand the course content ^b^ | √ | √ | √ | √ |
| *Cognitive Class/Lecture Engagement* |  |  |  |  |
| During a **typical [lecture/class]** in the last few weeks |  |  |  |  |
| I always summarized new [lecture/class] material in my own words when taking notes ^a^ | √ | √ | √ | √ |
| when I had difficulty understanding [lecture/class] material, I marked it to come back to later^a^ | x | √ | √ | √ |
| I focused on understanding the diagrams, charts, and figures presented in the [lecture/class] ^a^ | √ | √ | √ | √ |
| In this class I was able to stay focused and on-task____ of the time: |  |  |  |  |
| during [lab/recitation]. ^d^ | √ |  |  | x |
| during [lecture/class]. ^d^ | √ | x | x | x |
| while working on assignments. ^d^ | √ | x | x | x |
| *Cognitive Group Work Engagement* |  |  |  |  |
| During the latest group assignment: |  |  |  |  |
| I made sure to understand the plan for the project and my role in that plan^a^ | √ | x | √ |  |
| I was able to stay mentally focused while completing **my part of** the project ^a^ | √ | x | √ |  |

*Note*: Response options are indicated as follows: ^a^ Strongly disagree, Disagree, Agree, Strongly agree; ^b^ Never, Rarely, Often, Always; ^c^ Very difficult, Difficult, Easy, Very easy; ^d^ None, Some, Most, All, Not applicable

**Table B9**

*Dropped (x) or Loaded (√) Status of* *Behavioral* *Engagement Scale Items, Sorted by Space of Engagement*

| Stem and Item | General Chemistry 2 | Engineering Coding | Engineering Design | Genetics |
| --- | --- | --- | --- | --- |
| *Exam Studying* |  |  |  |  |
| While studying for the most recent exam or midterm: |  |  |  |  |
| other students quizzed me or asked for my help ^a^ | x | x |  |  |
| I spent _____ reorganizing my notes so the big ideas were clear ^b^ | √ | √ | √ | √ |
| *No stem was used in the following items* |  |  |  |  |
| I started studying for the most recent exam____ ^c^ | √ | √ | √ | √ |
| I spent __ hours studying alone for the most recent exam. ^d^ | √ | √ | √ | √ |
| I spent __ hours actively studying with classmates for the most recent exam. ^d^ | x | √ | x | x |
| I went to the instructor or TA's office hours while studying for the most recent exam __ ^e^ | x | x |  | x |
| *Behavioral Recitation Engagement* |  |  |  |  |
| I have attended _________ of the [recitations/labs] so far ^f^ | x |  |  | √ |
| I ________ worked with other students during [recitation/labs] ^g^ | x | x |  | x |
| I completed ______ of the activities we were given in [recitation/labs] ^f^ | √ |  |  | √ |
| *Behavioral Lecture/Class Engagement* |  |  |  |  |
| I have attended _____ of the course [lectures/classes] so far ^h^ | x | x |  | x |
| I _____ participated in the in-class activities during [lecture/class] (clicker response questions, hand raising, think-pair-share, etc.) ^i^ | x | x | x | x |
| *Behavioral Group Work Engagement* |  |  |  |  |
| I spent ___ hours with others on my team to complete the group project. ^d^ | √ | x | √ | x |
| I spent ___ hours on my own working on the team project. ^d^ | √ | x | √ | x |
| *Behavioral Assignment Engagement* |  |  |  |  |
| On the most difficult parts of each assignment, I ___^j^ | x | x |  |  |

*Note*: Response options are indicated as follows: ^a^ Never, Once, Rarely, Multiple times; ^b^ 0 minutes, 15 minutes, 30 minutes, 1 hour, 2 hours, 3 hours, 4+ hours; ^c^ the day of, the day before, several days before, a week or more before; ^d^ Numerical input ; ^e^ Never, Once, Twice, More than twice; ^f^ None, Some, Most, All, Not applicable; ^g^ Never, Seldom, Often, Always, Not applicable; ^h^ Some, Most, Almost all, All; ^i^ Never, Once, Rarely, Always; ^j^ Quickly took my best guess, Spent some time working on it, Spent much time working on it, Took as much time as needed

## Discussion

Similar to the prior studies, the emergent factorial structure was centered upon specific activity spaces of learning engagement. In addition, and reflecting the inclusion of new items, group assignment engagement emerged as a distinct factor. Also similar to the prior studies, the behavioral-cognitive distinction was very strong in the emergent structure, with each space of engagement loading only items from one mode. The other engagement items associated with each of those spaces failed to load on an underlying factor, most notably the four behavioral items related to lectures and recitations. Further, group-work produced separate cognitive and behavioral factors in the chemistry context, further supporting cognitive versus behavioral distinctions.

# Appendix C: Cognitive Interviews

To ensure the quality of the improved instrument, identify potential sources of response error, and enhance item clarity, the research team conducted a series of cognitive interviews with 15 participants drawn from a single, large, research-intensive, urban East coast institution in the United States. Combining criterion and stratified purposeful sampling (Trost, 1986), a diverse sample of participants were selected from students who had taken engineering, economics, or chemistry courses at the institution and consisted of five White, four Asian, four Multiracial, three Black, and one Latinx students. The sample was also diverse by way of gender, and consisted of seven women, seven men, one trans woman and one trans non-binary individual. Sample demographics are presented in Table C1. Following Beatty and Willis (2007) and Willis (2005), the interviews were conducted using a mixture of the think-aloud and probing processes following a structured protocol. Interviews lasted approximately 60-90 minutes and evaluated item clarity, interpretation, acceptability of the premises embedded in each item, and adequacy of response options. After data collection, the research team used *a priori* coding (Saldaña, 2021) to determine if the participant understood the items as intended by instrument designers, if there were misconceptions around item intent, if intent was adequately understood but phrasing could be improved, if the item was clear but the response options were inappropriate, or a combination of these responses. These data demonstrated that almost all participants accurately understood the intent of each item (see Table C2). Where they did not, participant reactions were used to further refine the phrasing of items and response options. For example, some respondents found the phrase “I flagged [lecture material] to come back to later” to be unclear or archaic, and suggested “I marked [lecture material]” instead. The finalized scale was then deployed for quantitative evaluation in chemistry and engineering contexts in the replication study and in engineering and biology contexts in the primary study.

**Table C1**

*Demographic Characteristics of Cognitive Interview Participants*

| Case | Gender | | | Race/Ethnicity | |
| --- | --- | --- | --- | --- | --- |
| Engineering students | |  | | |  |
| 1 | Man | | | Latinx | |
| 2 | Man | | | Black/Chicano | |
| 3 | Woman | | | White | |
| 4 | Woman | | | East Asian | |
| 5 | Man | | | White | |
| 6 | Nonbinary Trans* | | | Southeast Asian, Asian, White | |
| 7 | Nonbinary Trans* | | | East Asian | |
| Chemistry and Economics students | | |  | |  |
| 8 | Man | | | Black | |
| 9 | Woman | | | Black, American Indian | |
| 10 | Woman | | | Black | |
| 11 | Man | | | Latinx, Black | |
| 12 | Woman | | | White | |
| 13 | Woman | | | Indian/Pakistani | |
| 14 | Woman | | | Indian/Pakistani | |
| 15 | Man | | | White | |
| 16 | Man | | | White | |

**Table C2**

*Participant Responses from Engagement Scale Cognitive Interview*

| Item | Understood the item as intended | Understood the item but suggested … | | | Did not understand item/response options |
| --- | --- | --- | --- | --- | --- |
|  |  | Item phrasing revisions | Response option phrasing revisions | Phrasing revisions to both item and response options |  |
| Cognitive 4 | 11 | 1 | 2 | 0 | 2 |
| Cognitive 5 | 15 | 0 | 1 | 0 | 0 |
| Cognitive 6 | 15 | 0 | 1 | 0 | 0 |
| Cognitive 8 | 13 | 1 | 1 | 1 | 0 |
| Cognitive 9 | 12 | 3 | 1 | 0 | 0 |
| Cognitive 10 | 15 | 0 | 1 | 0 | 0 |
| Behavioral 1 | 13 | 0 | 3 | 0 | 0 |
| Behavioral 2 | 15 | 1 | 0 | 0 | 0 |
| Behavioral 3 | 14 | 0 | 2 | 0 | 0 |
| Behavioral 4 | 14 | 0 | 2 | 0 | 0 |
| Behavioral 5 | 15 | 0 | 1 | 0 | 0 |
| Behavioral 6 | 10 | 0 | 6 | 0 | 0 |
| Behavioral 7 | 15 | 0 | 1 | 0 | 0 |
| Behavioral 8 | 14 | 0 | 2 | 0 | 0 |
| Behavioral 9 | 15 | 0 | 1 | 0 | 0 |
| Behavioral 10 | 15 | 1 | 0 | 0 | 0 |
| Behavioral 11 | 13 | 3 | 0 | 0 | 0 |

# Appendix D: Scale Measurement Invariance Across Course Contexts

Ensuring the measurement invariance of psychometric instruments both across groups and over time is a critical concern for higher education scholars education (Litson & Feldon, 2022). To investigate the structural and metric invariance of the scale across various courses and disciplines—specifically chemistry, genetics, and two engineering courses at different institutions—as well as between first- and second-year STEM student populations (comparing genetics and chemistry), we conducted a series of statistical invariance tests. The investigation was driven by the following research questions: to what degree are common factors invariant in their measurement properties across disciplines? Are particular subscales more sensitive, more discriminating in some disciplines than in others? To what extent can measures of learning engagement in one discipline be compared to measures in others? And what does this reveal regarding the nature of engagement?

To address these questions and investigate the metric invariance of the scale, invariance analyses were conducted using multigroup structural equation modeling methods. These analyses reveal whether there are statistically significant differences in the measurement characteristics of each item and of the factors to which the items belong when administered in different contexts. While invariance indicates that both the scale behavior and the assessed phenomena have comparable characteristics despite context, significant non-invariance suggests several possibilities. For example, they may reflect differences in the nature of learning engagement across disciplines, variations in pedagogy and curriculum, context-sensitivity of the trait of learning engagement, or potentially the non-universality of certain aspects of learning engagement.

## Method

Multigroup SEM was used to estimate the measurement coefficients and standard errors of each item on their parent factor in each course or discipline. Item means were fixed to 0 and variances to 1 in all models, and the item measurement coefficients and other parameters were allowed to vary freely across groups. Subsequently, a Wald’s test was used to compare the item metrics across contexts to determine if the observed differences in item measurement characteristics were statistically meaningful (DeVellis, 2016). First, we examined item functioning across two different engineering courses (first-year engineering coding, *n* = 1116; first-year engineering design, *n* = 774) embedded in the same institutional context. Subsequently, we examined item functioning between two science courses (general chemistry 2, *n* = 401; genetics, *n* = 346) embedded in the same institutional context. Finally, we contrasted scale characteristics in engineering as a whole (coding and design, *n* = 1,890) to the sciences (general chemistry 2 and genetics, *n* = 747) in different institutional contexts.

## Results

The majority of factors and their constituent items demonstrated universal measurement properties in all deployed contexts, including those assessing in-exam focus, recitation engagement behaviors, cognitive engagement with group project work, and time spent on group projects. In contrast, exam studying and in-class cognition demonstrated several instances of measurement variance. For example, the item assessing how long one starts studying before the exam demonstrated a stronger relationship with the latent factor of exam studying in the first-year engineering coding course than the first-year engineering design course that preceded it. Similarly, when studying for an exam commences seems to play a much larger role in shaping exam studying behaviors in engineering overall than in the sciences. Contrastingly, studying behavior in the sciences appears to be more strongly shaped by the amount of time one spends studying with peers than studying behavior in engineering courses, potentially highlighting a more individual, less communal activity space for studying in this latter context. Table D1 summarizes the results of Wald’s test of metric invariance across each of the comparative contexts for each overall factor as well as for each item nested within them. Table D2 presents the measurement coefficients and standard errors of each item that showed significant non-invariance in each context.

**Table D1**

*Wald’s Test of Factor and Item Measurement Invariance Across Pairs of Contrast Courses*

|  | Engineering vs. Engineering | | Chemistry vs. Genetics | | Engineering vs. Sciences | |
| --- | --- | --- | --- | --- | --- | --- |
|  | $\chi^{2}$ | *p* | $\chi^{2}$ | *p* | $\chi^{2}$ | *p* |
| In-Exam Focus | 0.00 | .999 | 0.00^a^ | .999^a^ | 0.01 | .994 |
| C14: For the most recent test, it was easy to pay attention. | 0.00 | .999 | 0.00^a^ | .999^a^ | 0.00 | .999 |
| C15: For the most recent test, it was easy to think clearly. | 0.00 | .999 | 0.00^a^ | .999^a^ | 0.00 | .999 |
| Pre-Exam Studying | **17.47** | **.002** | 4.40 | .355 | **41.09** | **.000** |
| B2: While studying for the most recent exam or midterm I spent __ reorganizing my notes so the big ideas were clear. | 0.13 | .722 | 0.30 | .587 | 0.16 | .685 |
| B9: I started studying for the most recent exam [time categories]. | **13.80** | **.001** | 1.54 | .214 | **33.35** | **.000** |
| B10: I spent __ hours studying alone for the most recent exam. | 0.85 | .357 | 2.15 | .142 | 0.03 | .865 |
| B11: I spent __ hours actively studying with classmates for the most recent exam. | 3.27 | .071 | 0.01 | .935 | **5.19** | **.023** |
| Cognitive In-Class Engagement | 13.53 | .060 | 14.54 | .105 | **41.02** | **.000** |
| C4: During class, I asked clarifying questions about topics that were unclear. | 1.37 | .242 | 0.09 | .768 | 0.35 | .555 |
| C5: During class, I combined different pieces of information from the course in new ways (topics from different weeks, etc.). | **4.17** | **.041** | 0.10 | .757 | 0.46 | .496 |
| C6: During class, I made pictures, diagrams, charts, or other figures to help understand the course content. | 0.37 | .545 | 0.29 | .589 | 0.26 | .610 |
| C8: I always summarized new [**class**/**lecture**] material in my own words when taking notes. | 0.77 | .385 | 0.36 | .547 | **11.77** | **.001** |
| C9: When I had difficulty understanding [**class**/**lecture**] material, I marked it to come back to later. | **4.41** | **.036** | 1.84 | .175 | 1.74 | .188 |
| C10: I focused on understanding the diagrams, charts, and figures presented in the [**class**/**lecture**]. | 0.23 | .631 | 0.00 | .977 | **15.66** | **.000** |
| C16: I was able to stay focused and on-task__ of the time during recitation. |  |  | **9.28** | **.002** | 0.46 | .497 |
| C17: I was able to stay focused and on-task during class __ of the time. |  |  | **4.33** | **.037** | 0.72 | .395 |
| C18: I was able to stay focused and on-task__ of the time while working on assignments. | 2.40 | .122 | 3.01 | .083 | **7.66** | **.006** |
| Recitation Engagement |  |  | 0.00 | .999 | 0.00^b^ | .999^b^ |
| B3: I have attended __ of the recitations so far. |  |  | 0.00 | .999 | 0.00^b^ | .999^b^ |
| B5: I completed __ of the activities we were given in [**recitation**/**class**]. |  |  | 0.00 | .999 | 0.00^b^ | .999^b^ |
| Cognitive Group Project | 0.00 | .999 |  |  | 0.00 | .999 |
| C11: During the latest group assignment, I made sure to understand the plan for the project and my role in that plan. | 0.00 | .999 |  |  | 0.00 | .999 |
| C12: I was able to stay mentally focused while completing my part of the project. | 0.00 | .999 |  |  | 0.00 | .999 |
| Time on Group Project | 0.00 | .999 | 0.01 | .946 | -- ^c^ | -- ^c^ |
| B12: I spent ___ hours with others on my team to complete the group project. | 0.00 | .999 |  |  | -- ^c^ | -- ^c^ |
| B13: I spent ___ hours on my own working on the team project. | 0.00 | .998 | 0.01 | .946 | -- ^c^ | -- ^c^ |

*Note:* Shaded cells indicate item was not asked in one or both contexts. Non-invariant items are bolded. ^a^ Multigroup SEM models of these items failed to converge using traditional methods. Consequently, their outcomes were dichotomized (lowest 2 scores = 0, highest 2 scores = 1) for this analysis. ^b^ Indicates similar convergence challenges and dichotomization (highest score = 1, not highest score = 0). ^c^ SEM would not converge using a variety of integration methods or data transformations.

**Table D2**

*Measurement Coefficients of Non-Invariant Items Across Pairs of Contrast Courses*

|  | Engineering Coding  (Engineering Design) | | Chemistry  (Genetics) | | Engineering  (Sciences) | |
| --- | --- | --- | --- | --- | --- | --- |
| Item | Coef. | S.E. | Coef. | S.E. | Coef. | S.E. |
| B9: I started studying for the most recent exam [time categories]. | .693  (.550) | .028  (.026) | -- | -- | .629  (.410) | .019  (.032) |
| B11: I spent __ hours actively studying with classmates for the most recent exam. | -- | -- | -- | -- | .181  (.274) | .012  (.039) |
| C5: During class, I combined different pieces of information from the course in new ways (topics from different weeks, etc.). | .259  (.327) | .022  (.025) | -- | -- | -- | -- |
| C8: I always summarized new [**class**/**lecture**] material in my own words when taking notes. | -- | -- | -- | -- | .496  (.376) | .021  (.028) |
| C9: When I had difficulty understanding [**class**/**lecture**] material, I marked it to come back to later. | .523  (.436) | .026  (.032) | -- | -- | -- | -- |
| C10: I focused on understanding the diagrams, charts, and figures presented in the [**class**/**lecture**]. | -- | -- | -- | -- | .310  (.424) | .016  (.024) |
| C16: I was able to stay focused and on-task__ of the time during recitation. |  |  | .413  (.245) | .039  (.040) | -- | -- |
| C17: I was able to stay focused and on-task during class __ of the time. |  |  | .401  (.283) | .042  (.038) | -- | -- |
| C18: I was able to stay focused and on-task__ of the time while working on assignments. | -- | -- | -- | -- | .196  (.282) | .017  (.026) |

*Note:* Shaded cells indicate item was not asked in one or both contexts

## Discussion

A degree of measurement variance in cognitive in-class learning engagement is expected given the vastly differing pedagogies, focal skills, interactional environments, and curricula in varied STEM contexts; and while several in-class items were invariant across all contexts, no item was variant across all contexts, highlighting the nuance and variability of in-class learning experiences. For example, while the overall subscale was invariant between the two engineering courses, nonetheless engagement in the engineering coding classroom de-emphasized synthesizing ideas and placed more focus on identifying topics that were poorly understood than engagement in the engineering design class, potentially representing a shift in the centrality of these two metacognitive strategies as students progress through their first year of engineering education. Similarly, while the overall subscale was invariant across science courses, in-class and in-recitation focus played a larger role in learning in chemistry than in genetics contexts.

Finally, the in-class engagement subscale revealed overall differences between engineering and the sciences. Specifically, summarizing course material in one’s own words more powerfully shaped engineering class engagement, while comprehending diagrams, figures, and charts was more central to learning engagement in the sciences. These data reveal intriguing differences in what deep learning engagement looks like in different STEM courses and suggest potential avenues for further assessing and enhancing that engagement.
